# Supplementary material for: MAD2L2 promotes replication fork protection and recovery in a shieldin-independent and REV3L-dependent manner
Source: Nat Commun. 2022 Sep 8;13:5167. doi: 10.1038/s41467-022-32861-5 (PMC9458726; doi:10.1038/s41467-022-32861-5)
Supplement: Supplementary file 1 — Supplementary Information [file 41467_2022_32861_MOESM1_ESM.pdf]

Supplementary Information

MAD2L2 promotes replication fork protection and recovery in a shieldin-independent and REV3L-dependent manner

Inés Paniagua[1], Zainab Tayeh[1], Mattia Falcone[1], Santiago Hernández Pérez[1], Aurora Cerutti[1], Jacqueline J. L. Jacobs[1,*]

This file contains:

- Supplementary Fig. 1
- Supplementary Fig. 2
- Supplementary Fig. 3
- Supplementary Fig. 4
- Supplementary Fig. 5
- Supplementary Fig. 6
- Supplementary Fig. 7
- Supplementary References
- Extended Data 1

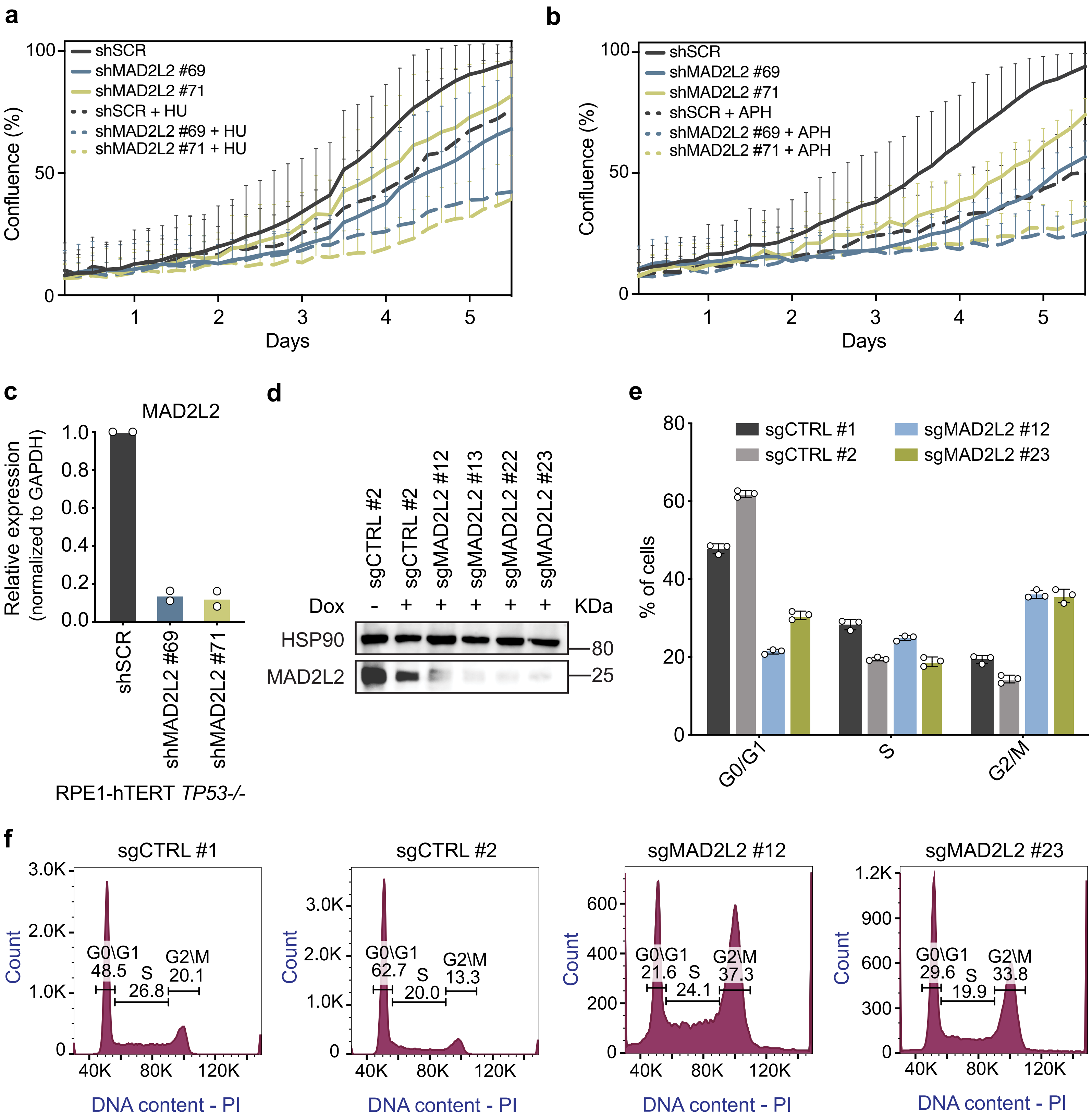

a
shSCR
shMAD2L2 #69
shMAD2L2 #71
shSCR + HU
shMAD2L2 #69 + HU
shMAD2L2 #71 + HU
Confluence (%)
Days
b
shSCR
shMAD2L2 #69
shMAD2L2 #71
shSCR + APH
shMAD2L2 #69 + APH
shMAD2L2 #71 + APH
Confluence (%)
Days
c
MAD2L2
Relative expression (normalized to GAPDH)
shSCR
shMAD2L2 #69
shMAD2L2 #71
RPE1-hTERT TP53-/-
d
sgCTRL #2
sgCTRL #2
sgMAD2L2 #12
sgMAD2L2 #13
sgMAD2L2 #22
sgMAD2L2 #23
Dox - + + + + +
KDa
HSP90
MAD2L2
80
25
e
sgCTRL #1
sgCTRL #2
sgMAD2L2 #12
sgMAD2L2 #23
% of cells
G0/G1
S
G2/M
f
sgCTRL #1
sgCTRL #2
sgMAD2L2 #12
sgMAD2L2 #23
Count
G0\G1 48.5
S 26.8
G2\M 20.1
G0\G1 62.7
S 20.0
G2\M 13.3
G0\G1 21.6
S 24.1
G2\M 37.3
G0\G1 29.6
S 19.9
G2\M 33.8
DNA content - PI

Supplementary Figure 1 - related to Fig. 1. Impaired DNA replication in MAD2L2-depleted cells is not associated with impaired S-phase progression

a, b, Proliferation of control (shSCR) and MAD2L2-depleted RPE1-hTERT *TP53*-/- cells, untreated (continuous line) or treated (dashed line) with 150 μM HU (**a**) or 0.2 μM aphidicolin (APH) (**b**). Confluency was measured using the Incucyte. Mean ± SD of three independent experiments is shown for (**a**), mean ± SEM from two independent experiments is shown for (**b**). **c,** qPCR analysis of *MAD2L2* expression in RPE1-hTERT *TP53*-/- cells. Data were normalized to *GAPDH* expression. Bars represent the mean. Each dot represents one of two independent experiments. **d,** Immunoblot analysis of MAD2L2 in HeLa cells stably expressing doxycycline-inducible sgRNAs, after 6 days of doxycycline treatment. HSP90 serves as loading control. A representative blot of three independent experiments is shown. **e,** Percentage of G1-, S- and G2-phase cells in control (sgCTRL) and MAD2L2-depleted HeLa cells, determined by propidium iodide (PI) staining and FACS analysis, after 6 days of doxycycline treatment. Of note, MAD2L2-depletion caused an increase in G2/M-phase cells, in line with a previously reported mitotic role for MAD2L2[1]. Bars represent the mean ± SD. Each dot represents one of three independent experiments. **f,** Example of the gating strategy for cell cycle analysis of the cells from Fig. S1e.

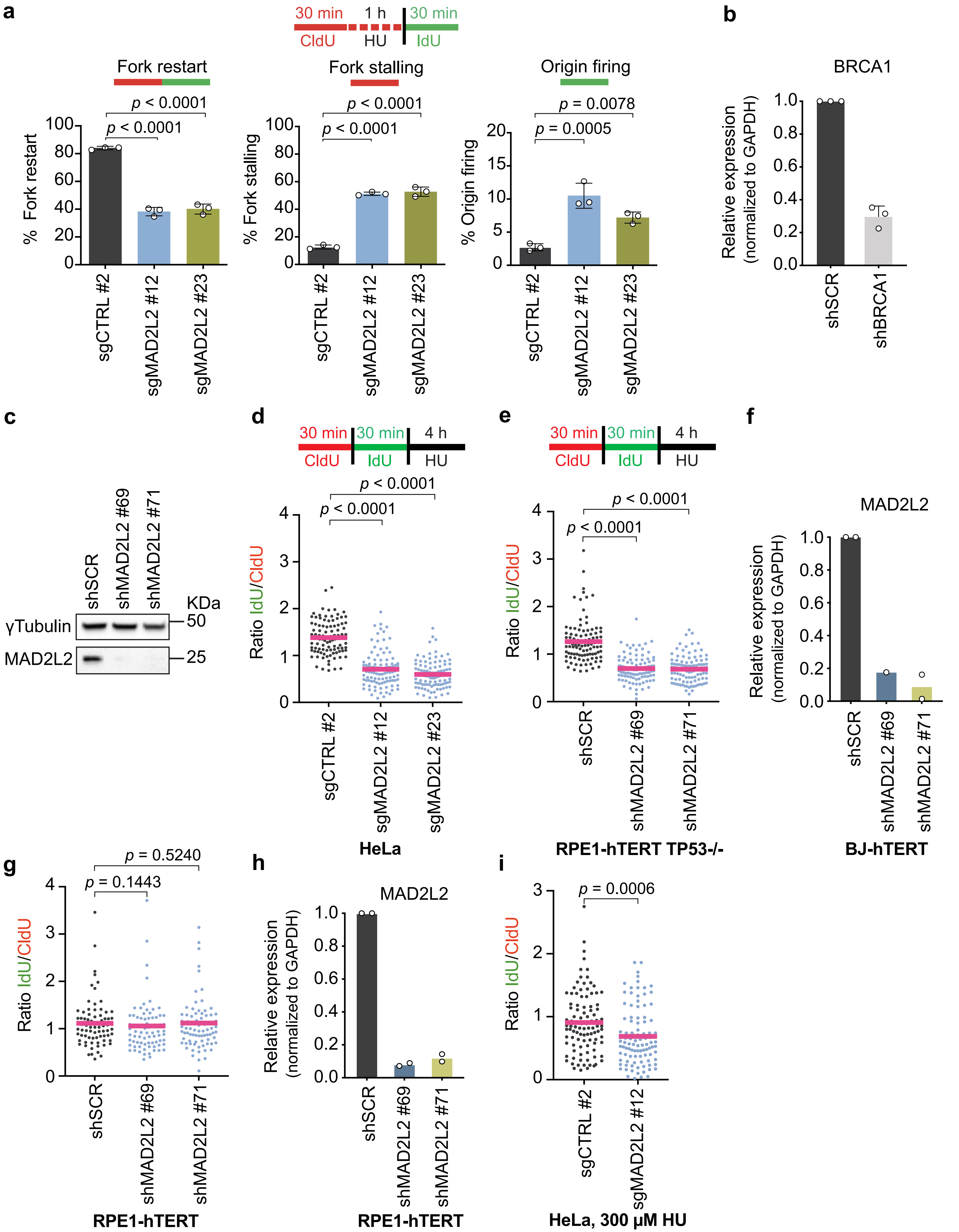
a
30 min
1 h
30 min
CldU
HU
IdU
Fork restart
Fork stalling
Origin firing
% Fork restart
% Fork stalling
% Origin firing
p < 0.0001
p < 0.0001
p = 0.0078
p = 0.0005
sgCTRL #2
sgMAD2L2 #12
sgMAD2L2 #23
b
BRCA1
Relative expression
(normalized to GAPDH)
shSCR
shBRCA1
c
shSCR
shMAD2L2 #69
shMAD2L2 #71
KDa
γTubulin
MAD2L2
50
25
d
30 min
30 min
4 h
CldU
IdU
HU
Ratio IdU/CldU
HeLa
e
shSCR
shMAD2L2 #69
shMAD2L2 #71
RPE1-hTERT TP53-/-
f
MAD2L2
BJ-hTERT
g
p = 0.5240
p = 0.1443
RPE1-hTERT
h
MAD2L2
RPE1-hTERT
i
p = 0.0006
sgCTRL #2
sgMAD2L2 #12
HeLa, 300 μM HU

Supplementary Figure 2 - related to Fig. 2. MAD2L2 protects stalled forks and promotes fork restart

a, Schematic and quantification of replication fork restart assays. CldU pulse-labeled forks under 1 h HU treatment (4 mM) were measured in control (sgCTRL) and MAD2L2-depleted HeLa cells. Bars represent the mean ± SD of three independent experiments. **b,** qPCR analysis of *BRCA1* expression in cells from Fig. 2e. Data were normalized to *GAPDH* expression. Bars represent the mean ± SD. Each dot represents one of three independent experiments. **c,** Immunoblot analysis of MAD2L2 in HeLa cells transduced with the indicated shRNAs from Fig. 2e. γ-Tubulin serves as loading control. A representative blot of three independent experiments is shown. **d,** Schematic and quantification of fork degradation assays in control (sgCTRL) and MAD2L2-depleted HeLa cells. Cells were treated with 4 mM HU for 4 h before being harvested. **e,** Schematic and quantification of fork degradation assays in control (shSCR) and MAD2L2-depleted RPE1 hTERT *TP53*-/- cells. Cells were treated as indicated above (**d**). **f**, qPCR analysis of *MAD2L2* expression in cells from Fig. 2f. Data were normalized to *GAPDH* expression. Bars represent the mean of two independent experiments. **g,** Schematic and quantification of fork degradation assays in control (shSCR) and MAD2L2-depleted RPE1-hTERT cells. Cells were treated as indicated above (**d**). **h,** qPCR analysis of *MAD2L2* expression in cells from Fig. S2g. Data were normalized to *GAPDH* expression. Bars represent the mean. Each dot represents one of two independent experiments. **i**, Quantification of fork degradation assays in control (sgCTRL) and MAD2L2-depleted HeLa cells treated with 300 μM HU for 4 h before being harvested. A representative fiber experiment from three independent biological replicates is shown for (**e, i**). A representative fiber experiment from two independent biological replicates is shown for (**d, g**). Pink bars in fiber plots represent the mean. Statistical analysis for the fiber assays in (**d, e, g, i**) was performed according to two-tailed Mann-Whitney test. Additional replicates and combined fiber plots are depicted in Extended Data 1 (or for Fig. S2d, an additional replicate is shown in Fig. S3f).

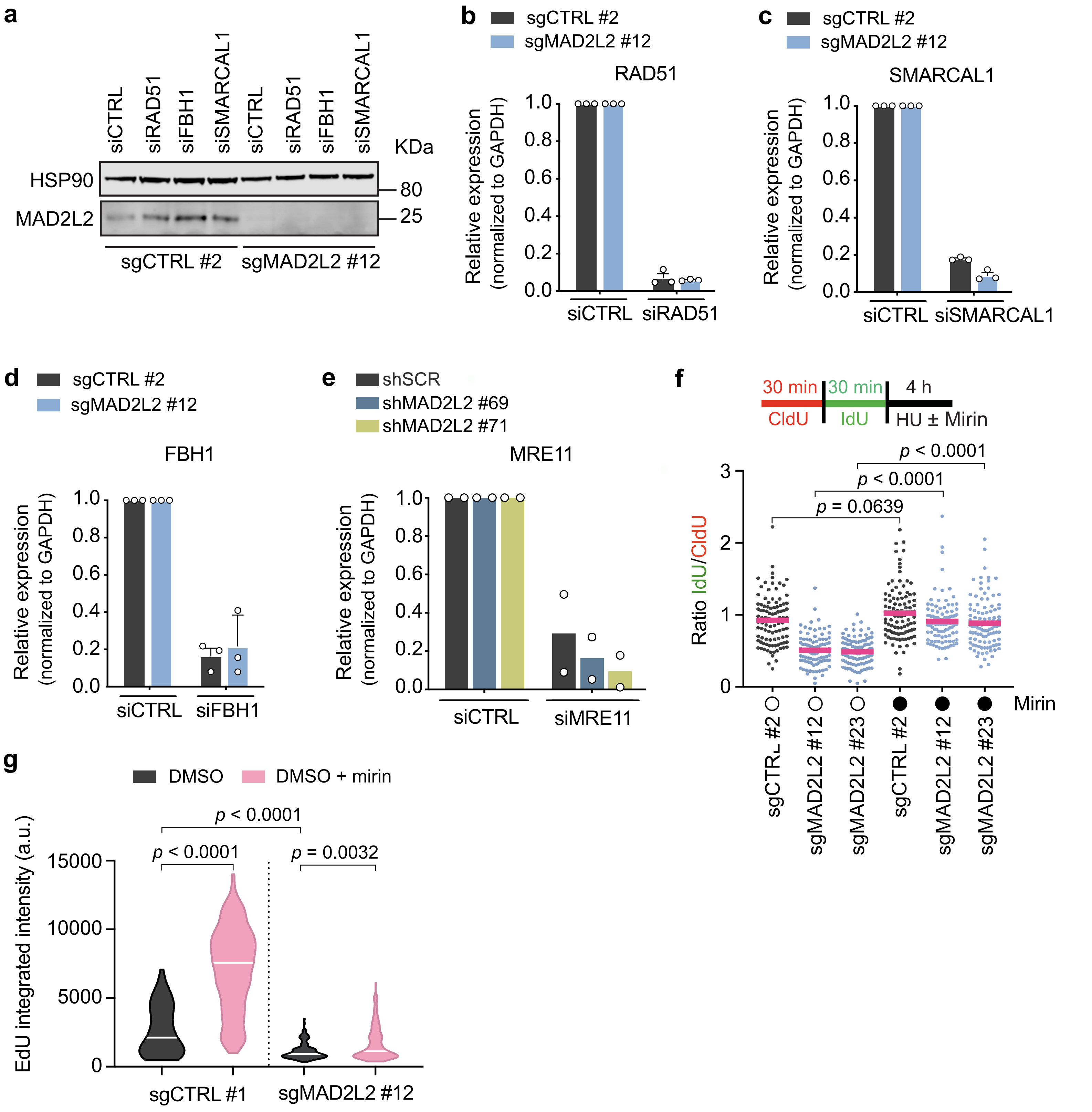

a
siCTRL
siRAD51
siFBH1
siSMARCAL1
siCTRL
siRAD51
siFBH1
siSMARCAL1
KDa
HSP90
80
MAD2L2
25
sgCTRL #2
sgMAD2L2 #12
b
sgCTRL #2
sgMAD2L2 #12
RAD51
Relative expression (normalized to GAPDH)
1.0
0.8
0.6
0.4
0.2
0.0
siCTRL
siRAD51
c
sgCTRL #2
sgMAD2L2 #12
SMARCAL1
Relative expression (normalized to GAPDH)
siCTRL
siSMARCAL1
d
sgCTRL #2
sgMAD2L2 #12
FBH1
Relative expression (normalized to GAPDH)
siCTRL
siFBH1
e
shSCR
shMAD2L2 #69
shMAD2L2 #71
MRE11
Relative expression (normalized to GAPDH)
siCTRL
siMRE11
f
30 min
30 min
4 h
CldU
IdU
HU ± Mirin
p < 0.0001
p < 0.0001
p = 0.0639
Ratio IdU/CldU
3
2
1
0
Mirin
sgCTRL #2
sgMAD2L2 #12
sgMAD2L2 #23
sgCTRL #2
sgMAD2L2 #12
sgMAD2L2 #23
g
DMSO
DMSO + mirin
p < 0.0001
p < 0.0001
p = 0.0032
EdU integrated intensity (a.u.)
15000
10000
5000
0
sgCTRL #1
sgMAD2L2 #12

Supplementary Figure 3 - related to Fig. 3. Inhibition of MRE11 and inactivation of RAD51 or SMARCAL1, but not FBH1, rescue fork degradation in MAD2L2-depleted cells

a, Immunoblot analysis of MAD2L2 in cells from Fig. 3a. HSP90 serves as loading control. A representative blot of three independent experiments is shown. **b, c, d,** qPCR analysis of *RAD51* (**b**), *SMARCAL1* (**c**), and *FBH1* (**d**) expression in cells from Fig. 3a. Data were normalized to *GAPDH* expression. Bars represent the mean ± SD. Each dot represents one of three independent experiments. **e**, qPCR analysis of *MRE11* expression in cells from Fig. 3b. Data were normalized to *GAPDH* expression. Bars represent the mean. Each dot represents one of two independent experiments. **f,** Schematic and quantification of fork degradation assays. Control (sgCTRL) and MAD2L2-depleted HeLa cells were treated with 4 mM HU ± 50 μM mirin for 4 h. Open circles, no treatment; closed circles, treatment. A representative fiber experiment from two independent biological replicates is shown (with another replicate in Fig. S2d). **g,** Assessment of DNA synthesis rates in control and MAD2L2-depleted HeLa cells following treatment with or without 50 μM mirin for 2 h. White bars represent the median of two independent experiments (each with 200 cells per condition). Statistical analysis was performed according to one-way Anova with Holm-Šidák's multiple comparison test. Pink bars in fiber plots represent the mean. Statistical analysis for the fiber assays in (**f**) was performed according to two-tailed Mann-Whitney test.

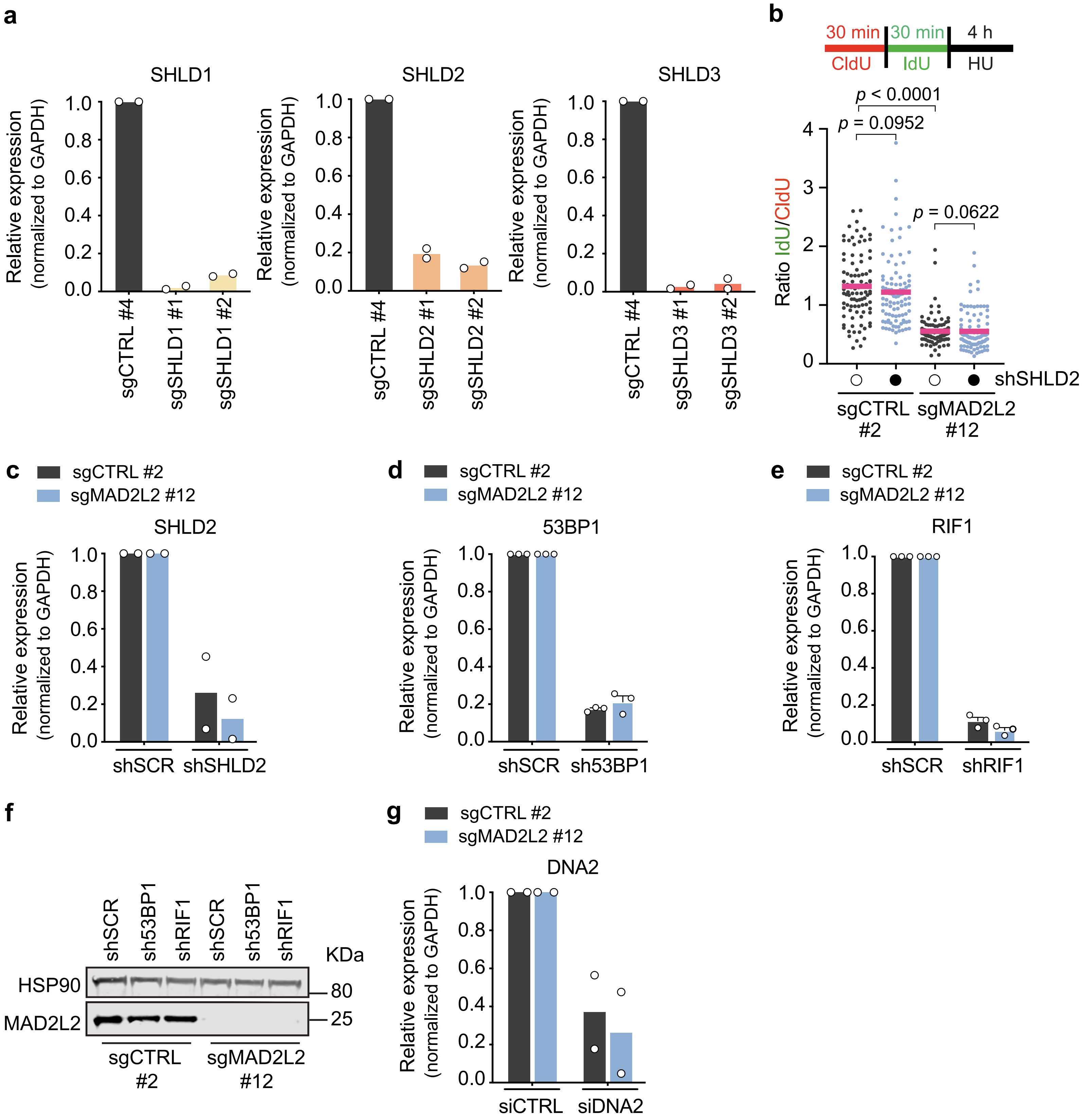

a
SHLD1
SHLD2
SHLD3
Relative expression (normalized to GAPDH)
1.0
0.8
0.6
0.4
0.2
0.0
sgCTRL #4
sgSHLD1 #1
sgSHLD1 #2
sgSHLD2 #1
sgSHLD2 #2
sgSHLD3 #1
sgSHLD3 #2
b
30 min
30 min
4 h
CldU
IdU
HU
Ratio IdU/CldU
p < 0.0001
p = 0.0952
p = 0.0622
shSHLD2
sgCTRL #2
sgMAD2L2 #12
c
sgCTRL #2
sgMAD2L2 #12
SHLD2
shSCR
shSHLD2
d
53BP1
sh53BP1
e
RIF1
shRIF1
f
shSCR
sh53BP1
shRIF1
KDa
HSP90
MAD2L2
80
25
sgCTRL #2
sgMAD2L2 #12
g
DNA2
siCTRL
siDNA2

Supplementary Figure 4 - related to Fig. 4. MAD2L2 suppresses degradation of stalled replication forks independently from shieldin and its upstream recruiter 53BP1/RIF1 at DSBs

a, qPCR analysis of *SHLD1, SHLD2* and *SHLD3* expression in HeLa cells from Fig. 4a-d. Data were normalized to *GAPDH* expression. Bars represent the mean. Each dot represents one of two independent experiments. See Supplementary Table 3 for gene-editing efficiencies of the sgRNAs. **b,** Schematic and quantification of fork degradation assays in control (sgCTRL) and MAD2L2-depleted HeLa cells transduced with a shRNA targeting SHLD2. Cells were treated with 4 mM HU for 4 h before being harvested. Open circles, control shRNA; closed circles, SHLD2 shRNA. A representative fiber experiment from two independent biological replicates is shown. Pink bars in fiber plots represent the mean. Statistical analysis for the fiber assays was performed according to two-tailed Mann-Whitney test. Additional replicates and combined fiber plots are depicted in Extended Data 1. **c,** qPCR analysis of *SHLD2* expression in cells from Fig. S4b. Bars represent the mean. Each dot represents one of two independent experiments. **d, e**, qPCR analysis of *53BP1* (**d**) or *RIF1* (**e**) expression in cells from Fig. 4e. Bars represent the mean ± SD. Each dot represents one of three independent experiments. **f,** Immunoblot analysis of MAD2L2 in cells from Fig. 4e. HSP90 serves as loading control. A representative blot of three independent experiments is shown. **g**, qPCR analysis of *DNA2* expression in cells from Fig. 4f. Bars represent the mean. Each dot represents one of two independent experiments.

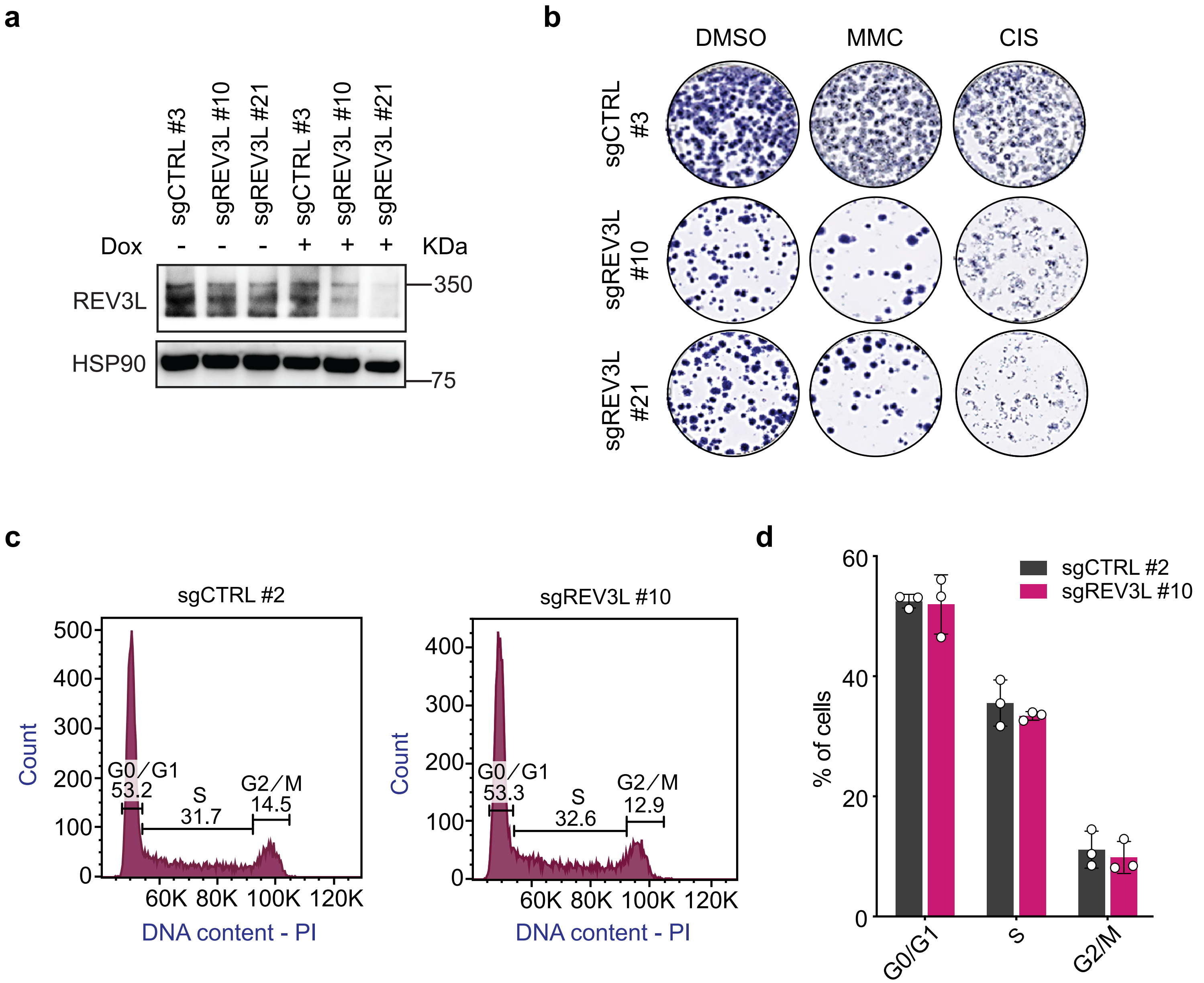
a
sgCTRL #3
sgREV3L #10
sgREV3L #21
sgCTRL #3
sgREV3L #10
sgREV3L #21
Dox
KDa
REV3L
HSP90
350
75
b
DMSO
MMC
CIS
sgCTRL #3
sgREV3L #10
sgREV3L #21
c
sgCTRL #2
sgREV3L #10
Count
G0/G1 53.2
S 31.7
G2/M 14.5
G0/G1 53.3
S 32.6
G2/M 12.9
DNA content - PI
d
sgCTRL #2
sgREV3L #10
% of cells
G0/G1
S
G2/M

Supplementary Figure 5 - related to Fig. 5. Validation of REV3L loss and assessment of cell cycle distribution in conditional *REV3L* knockout cells

a, Immunoblot analysis of REV3L in HeLa cells stably expressing doxycycline-inducible sgRNAs, after 6 days of doxycycline treatment. HSP90 serves as loading control. A representative blot of three independent experiments is shown. **b,** Representative images of survival assay in cells from Fig 5a. **c,** Example of the gating strategy for cell cycle analysis performed on control (sgCTRL) and REV3L-depleted HeLa cells and shown in (**d**). **d,** Percentage of G1-, S- and G2-phase cells, determined by propidium iodide (PI) staining and FACS analysis, after 6 days of doxycycline treatment. Bars represent the mean ± SD. Each dot represents one of three independent experiments.

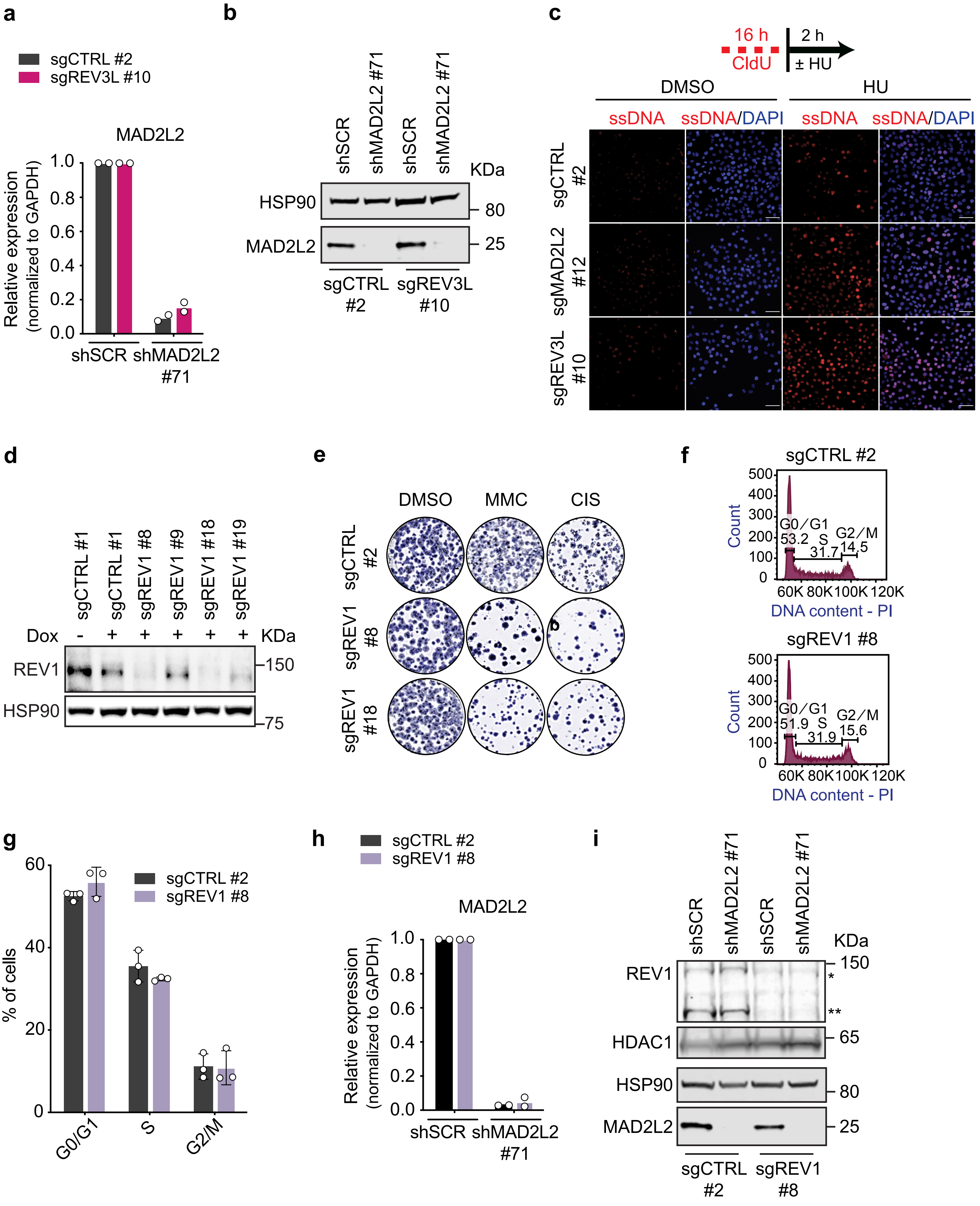

a
sgCTRL #2
sgREV3L #10
MAD2L2
Relative expression (normalized to GAPDH)
shSCR
shMAD2L2 #71
b
shSCR
shMAD2L2 #71
KDa
HSP90
MAD2L2
80
25
sgCTRL #2
sgREV3L #10
c
16 h
CldU
2 h
± HU
DMSO
HU
ssDNA
ssDNA/DAPI
sgCTRL #2
sgMAD2L2 #12
sgREV3L #10
d
sgCTRL #1
sgREV1 #8
sgREV1 #9
sgREV1 #18
sgREV1 #19
Dox
REV1
HSP90
150
75
e
DMSO
MMC
CIS
sgCTRL #2
sgREV1 #8
sgREV1 #18
f
sgCTRL #2
Count
G0/G1
53.2
S
31.7
G2/M
14.5
DNA content - PI
sgREV1 #8
51.9
31.9
15.6
g
% of cells
sgCTRL #2
sgREV1 #8
G0/G1
S
G2/M
h
sgCTRL #2
sgREV1 #8
MAD2L2
Relative expression (normalized to GAPDH)
shSCR
shMAD2L2 #71
i
shSCR
shMAD2L2 #71
KDa
REV1
HDAC1
HSP90
MAD2L2
150
65
80
25
sgCTRL #2
sgREV1 #8

Supplementary Figure 6 - related to Fig. 6. MAD2L2 protects stalled replication forks and prevents ssDNA accumulation in a REV3L- and REV1-dependent manner

a, qPCR analysis of *MAD2L2* expression in cells from Fig. 6a. Data were normalized to *GAPDH* expression. Bars represent the mean. Each dot represents one of two independent experiments. **b,** Immunoblot analysis of MAD2L2 in cells from Fig. 6a. HSP90 serves as loading control. A representative blot of two independent experiments is shown. **c,** Schematic and representative images of ssDNA-positive cells from Fig. 6b. Following initial labeling with CldU (red) for 16 h, cells were treated with or without 4 mM HU for 2 h. Scale bar, 50 μM. Quantification of three biological replicates is in (**6b**). **d,** Immunoblot analysis of REV1 in HeLa cells stably expressing doxycycline-inducible sgRNAs, after 6 days of doxycycline treatment. HSP90 serves as loading control. A representative blot of three independent experiments is shown. **e,** Representative images of survival assays in cells from Fig. 6c. **f,** Example of the gating strategy for cell cycle analysis performed on control (sgCTRL) and REV1-depleted HeLa cells and shown in (**g**). **g,** Percentage of G1-, S- and G2-phase cells, determined by propidium iodide (PI) staining and FACS analysis, after 6 days of doxycycline treatment. **h,** qPCR analysis of *MAD2L2* expression in cells from Fig. 6h. Data were normalized to *GAPDH* expression. Bars represent the mean. Each dot represents one of two independent experiments. **i,** Immunoblot analysis of MAD2L2 and REV1 in cells from Fig. 6h. HDAC1 and HSP90 serve as loading controls. A representative blot of two independent experiments is shown. Asterisk indicates a non-specific band running just below endogenous REV1, double asterisk indicates a degradation or alternative REV1 product that (next to full-length REV1) is lost upon CRISPR knockout of REV1.

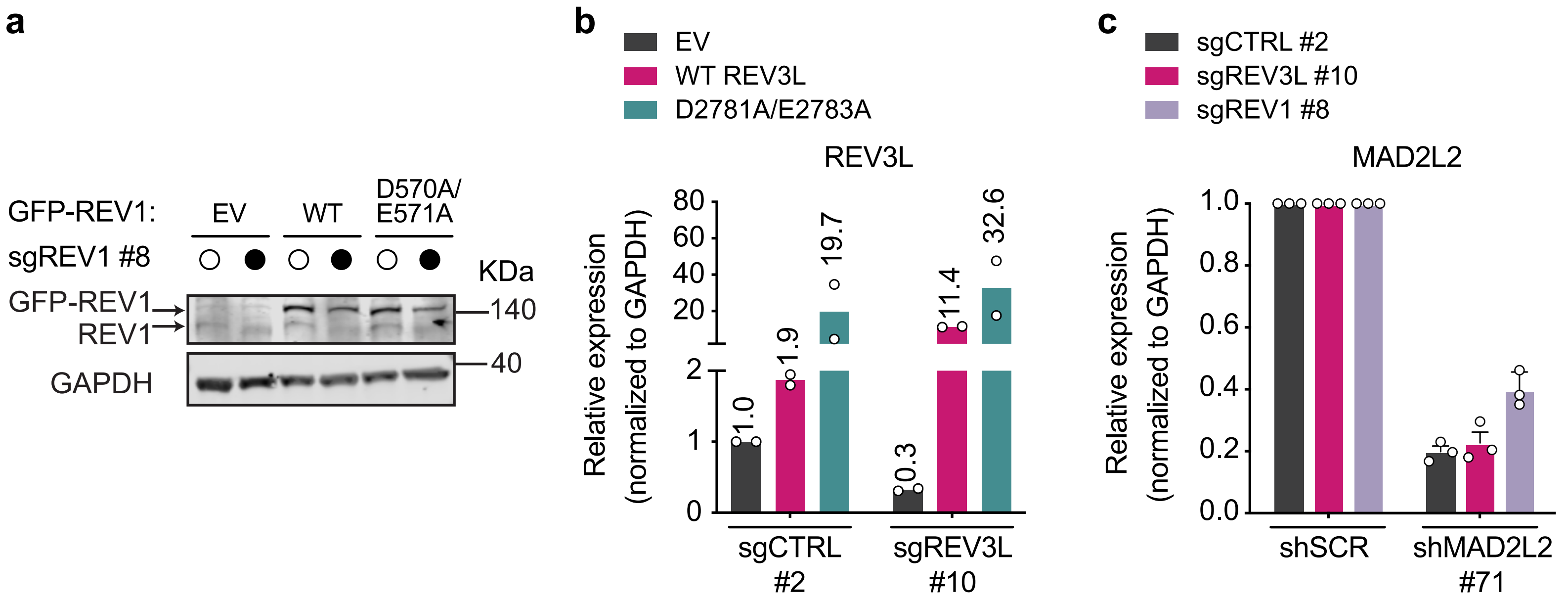
a
GFP-REV1:
EV
WT
D570A/
E571A
sgREV1 #8
KDa
GFP-REV1
REV1
140
GAPDH
40
b
EV
WT REV3L
D2781A/E2783A
REV3L
Relative expression
(normalized to GAPDH)
80
60
40
20
2
1
0
1.0
1.9
19.7
0.3
11.4
32.6
sgCTRL
#2
sgREV3L
#10
c
sgCTRL #2
sgREV3L #10
sgREV1 #8
MAD2L2
Relative expression
(normalized to GAPDH)
1.0
0.8
0.6
0.4
0.2
0.0
shSCR
shMAD2L2
#71

Supplementary Figure 7 - related to Fig. 7. Expression of wildtype or mutant REV1/REV3L and depletion of MAD2L2 in REV1/REV3L knockout cells to assess fork degradation and chromosomal instability

a, Immunoblot analysis of REV1 in HeLa cells from Fig. 7a. GAPDH serves as loading control. A representative blot of three independent experiments is shown. **b**, qPCR analysis of *REV3L* expression in cells from Fig. 7b. Data were normalized to GAPDH expression. Bars represent the mean. Each dot represents one of two independent experiments. **c**, qPCR analysis of *MAD2L2* expression in cells from Fig. 7c. Data were normalized to GAPDH expression. Bars represent the mean ± SD. Each dot represents one of three independent experiments.

Supplementary References

1 Bhat, A., Wu, Z., Maher, V. M., McCormick, J. J. & Xiao, W. Rev7/Mad2B plays a critical role in the assembly of a functional mitotic spindle. *Cell Cycle* **14**, 3929-3938, doi:10.1080/15384101.2015.1120922 (2015).

Extended Data 1: Additional fiber replicates and combined fiber plots of all replicates

Extended Data 1a - related to Fig. 2c

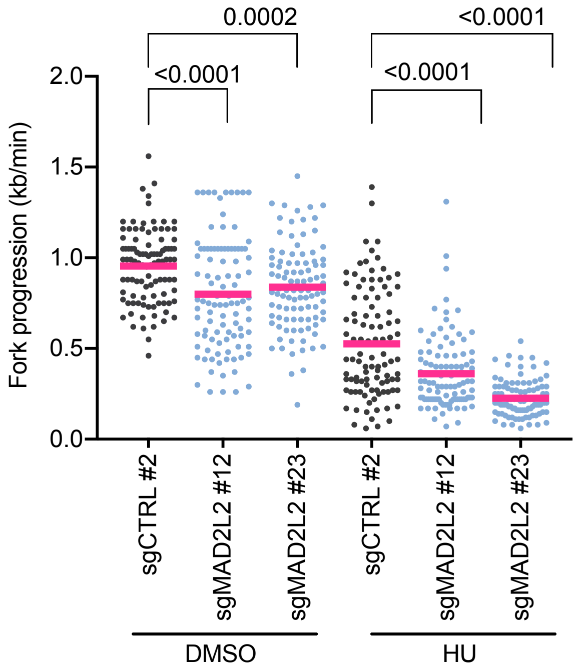

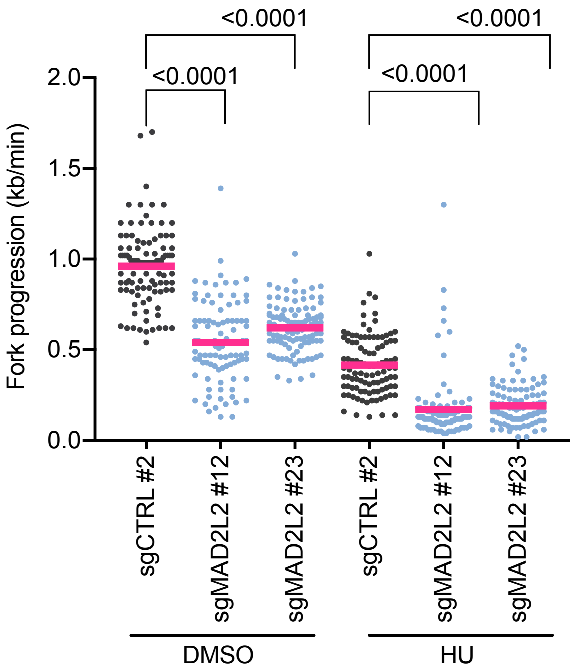

Additional replicates

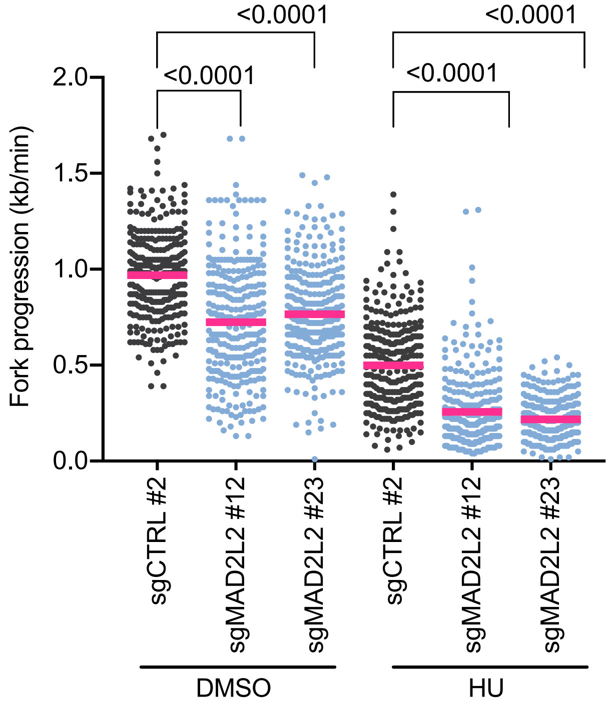

All replicates combined

Extended Data 1: Additional fiber replicates and combined fiber plots of all replicates

Extended Data 1b - related to Fig. 2e

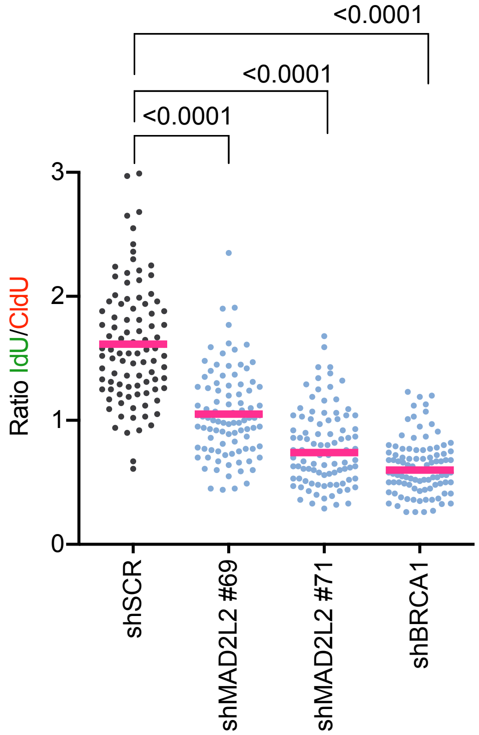

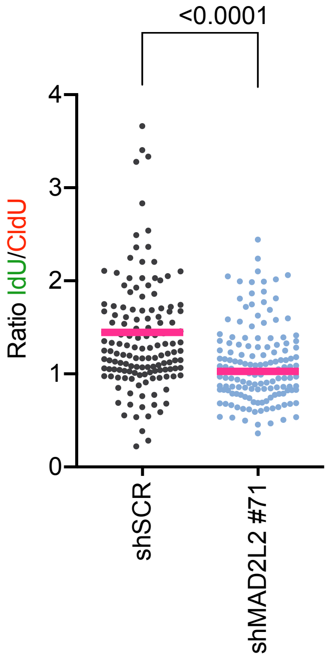

Additional replicates

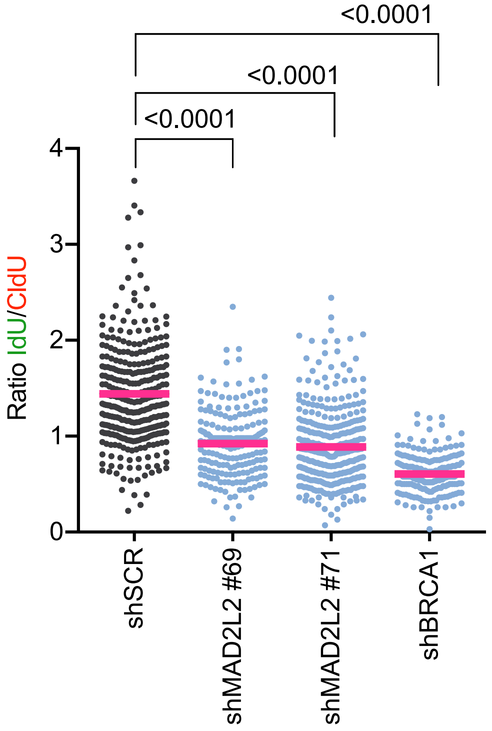

All replicates combined

Extended Data 1: Additional fiber replicates and combined fiber plots of all replicates

Extended Data 1c - related to Fig. 2f

BJ-hTERT

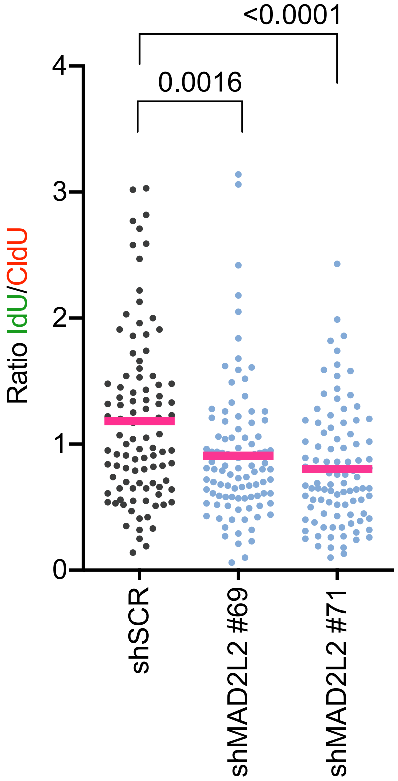

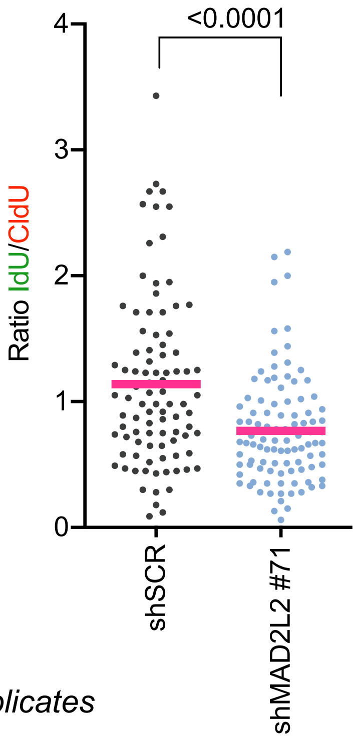

Additional replicates

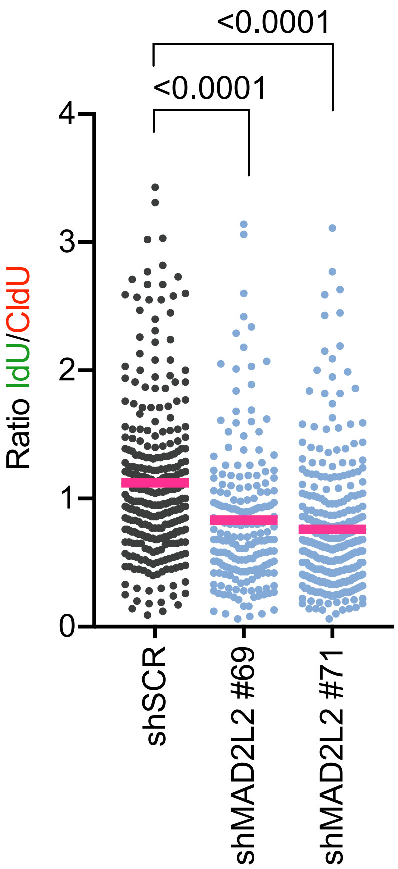

All replicates combined

Extended Data 1: Additional fiber replicates and combined fiber plots of all replicates

Extended Data 1d - related to Fig. 2i

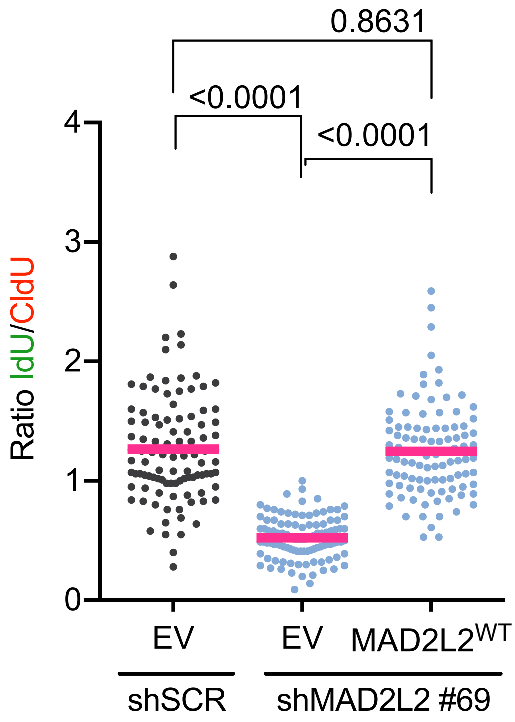

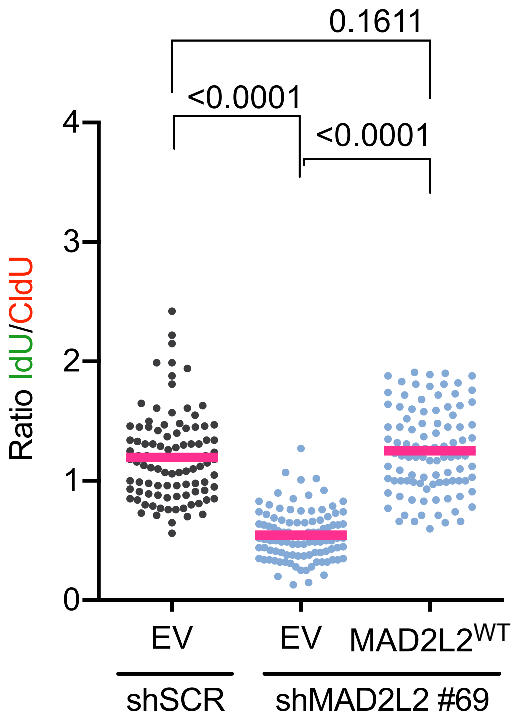

Additional replicates

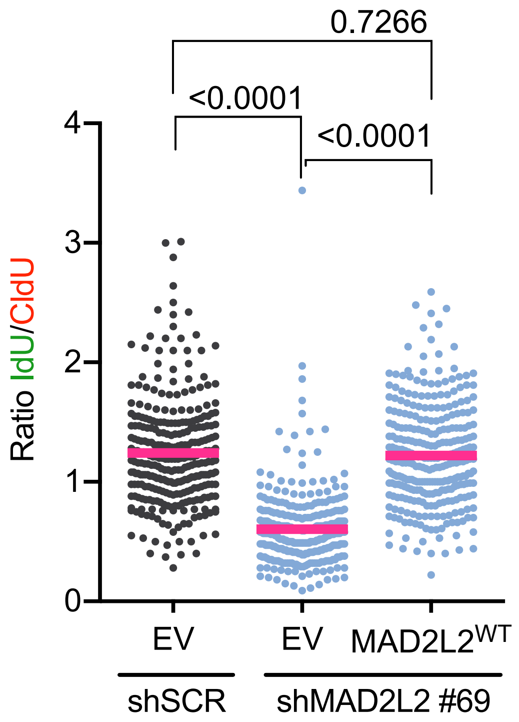

All replicates combined

Extended Data 1: Additional fiber replicates and combined fiber plots of all replicates

Extended Data 1e - related to Fig. S2f

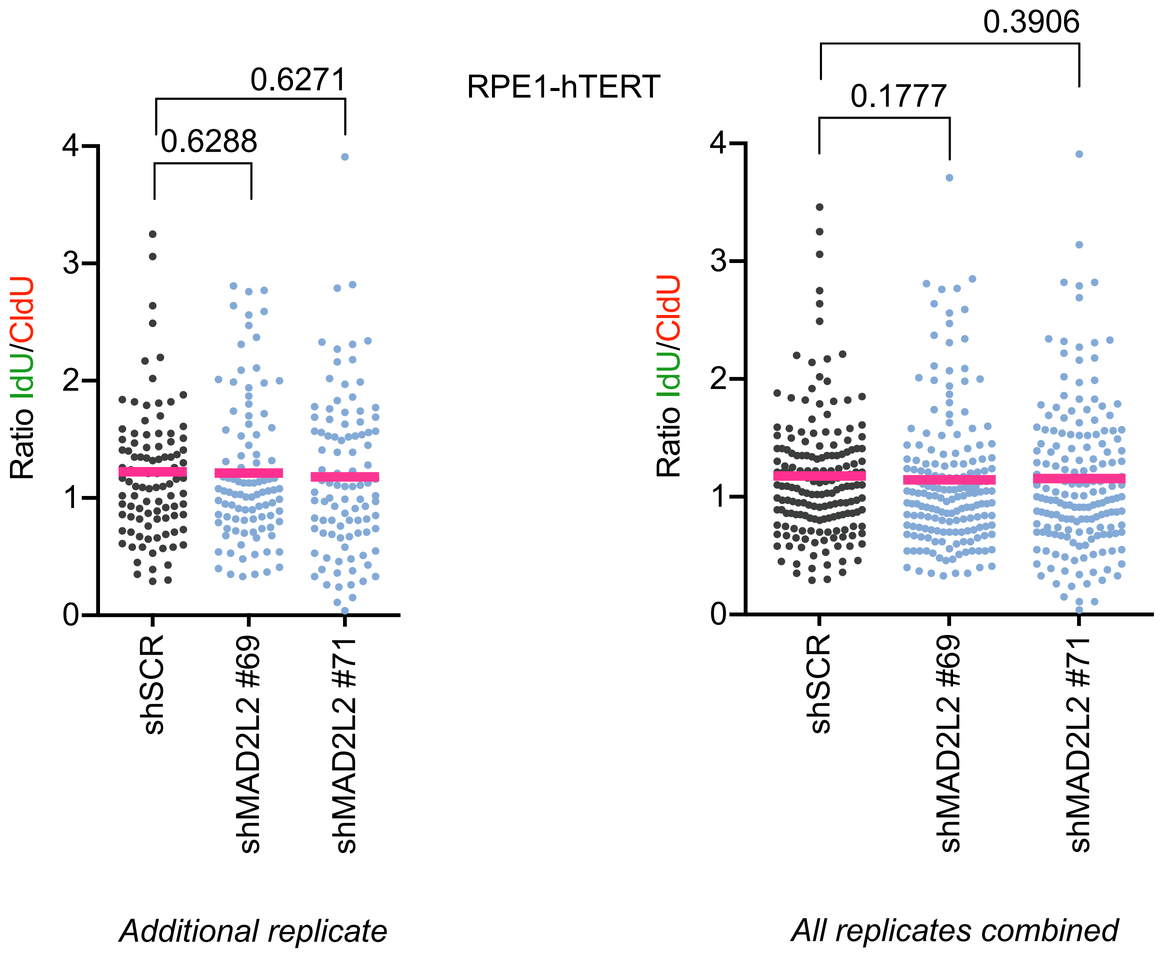

Extended Data 1: Additional fiber replicates and combined fiber plots of all replicates

Extended Data 1f - related to Fig. S2h

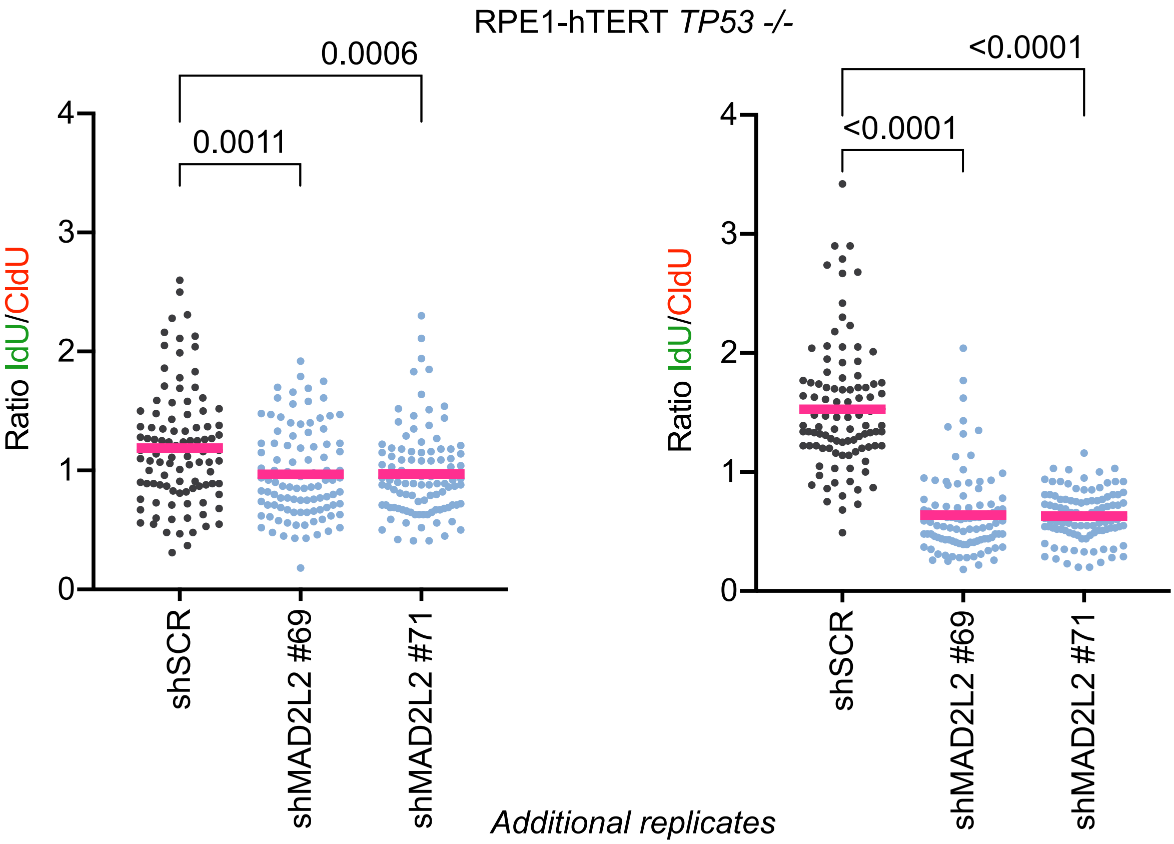

Additional replicates

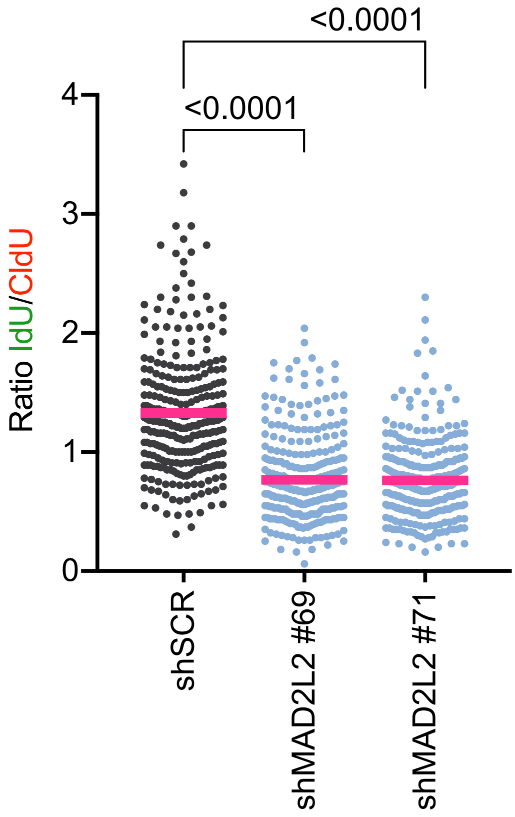

All replicates combined

Extended Data 1: Additional fiber replicates and combined fiber plots of all replicates

Extended Data 1g - related to Fig. S2i

HeLa, 300 μM HU

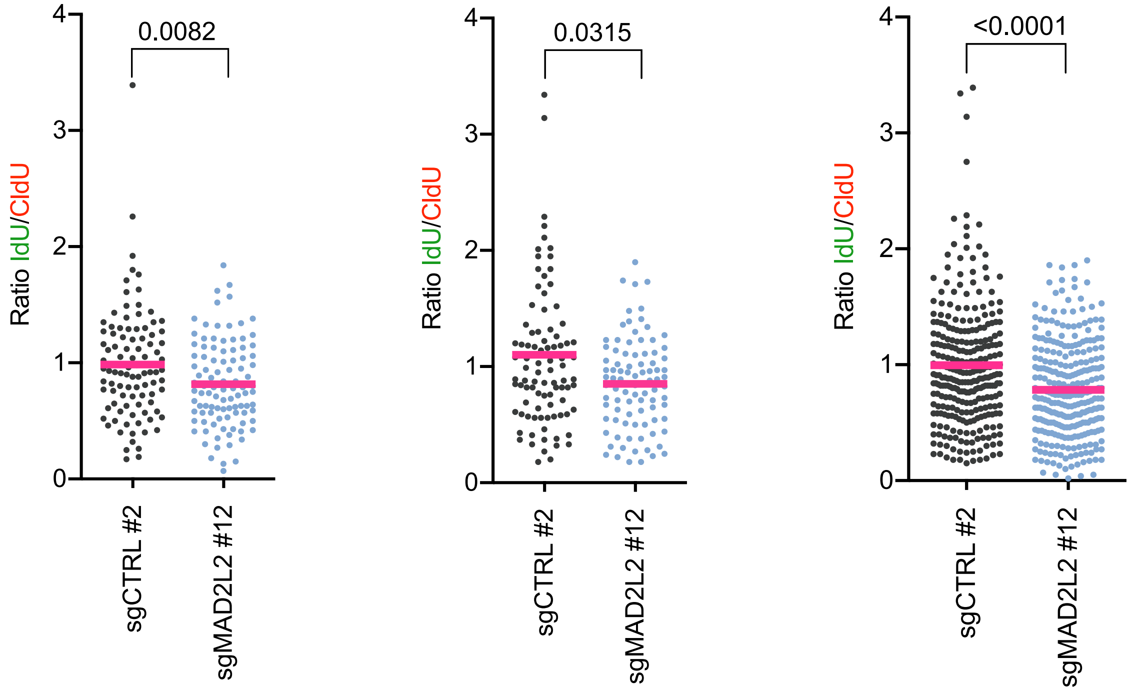

Additional replicates

All replicates combined

Extended Data 1: Additional fiber replicates and combined fiber plots of all replicates

Extended Data 1h - related to Fig. 3a

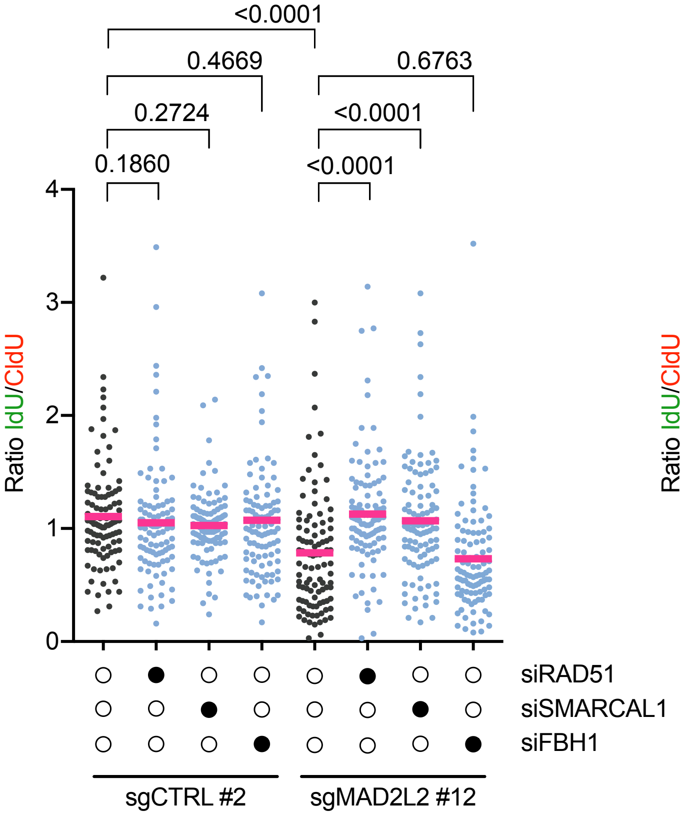

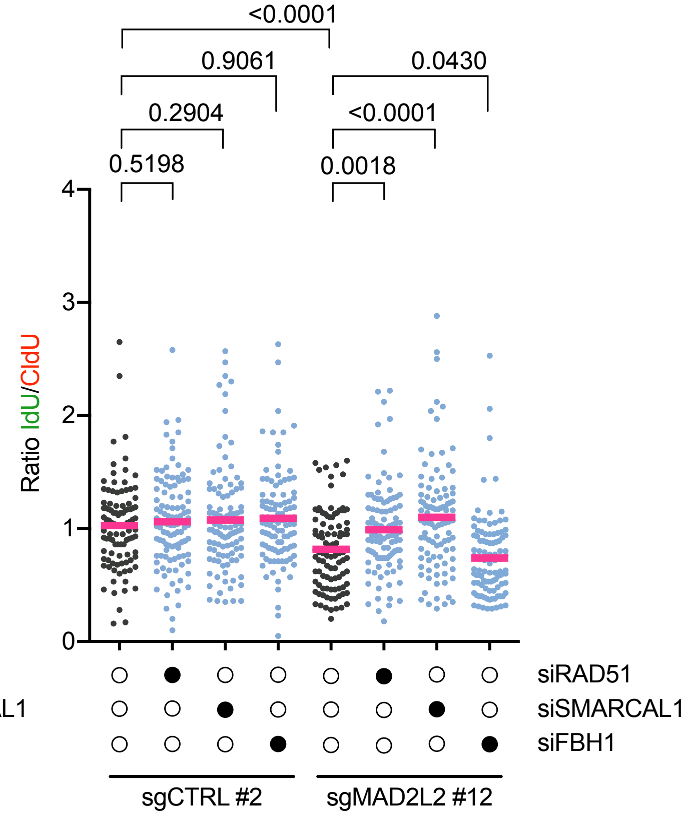

Additional replicates

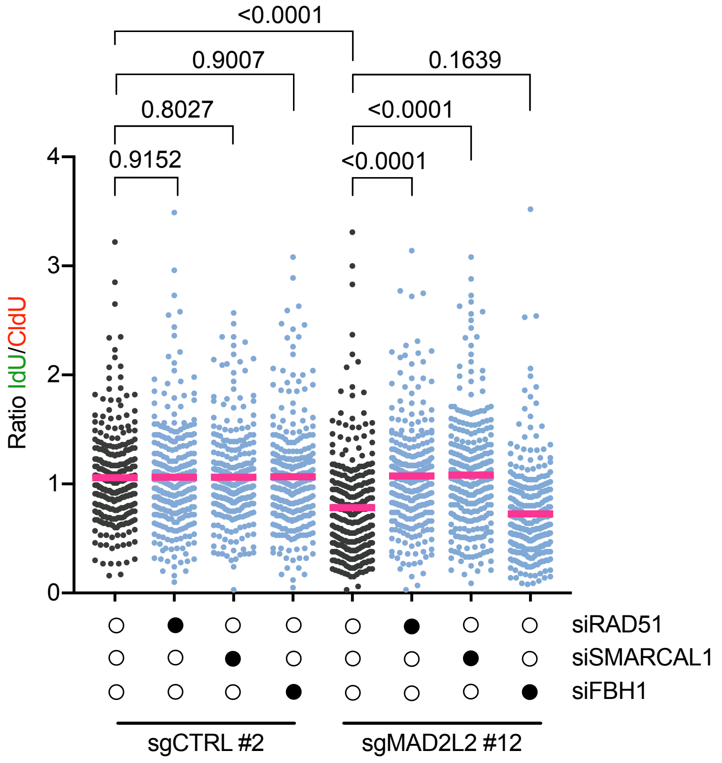

All replicates combined

Extended Data 1: Additional fiber replicates and combined fiber plots of all replicates

Extended Data 1i - related to Fig. 3b

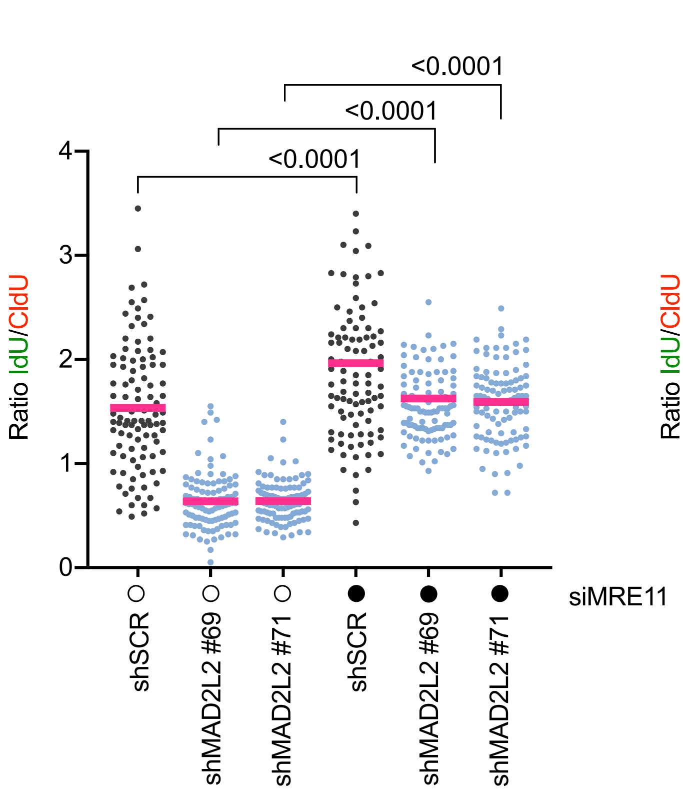

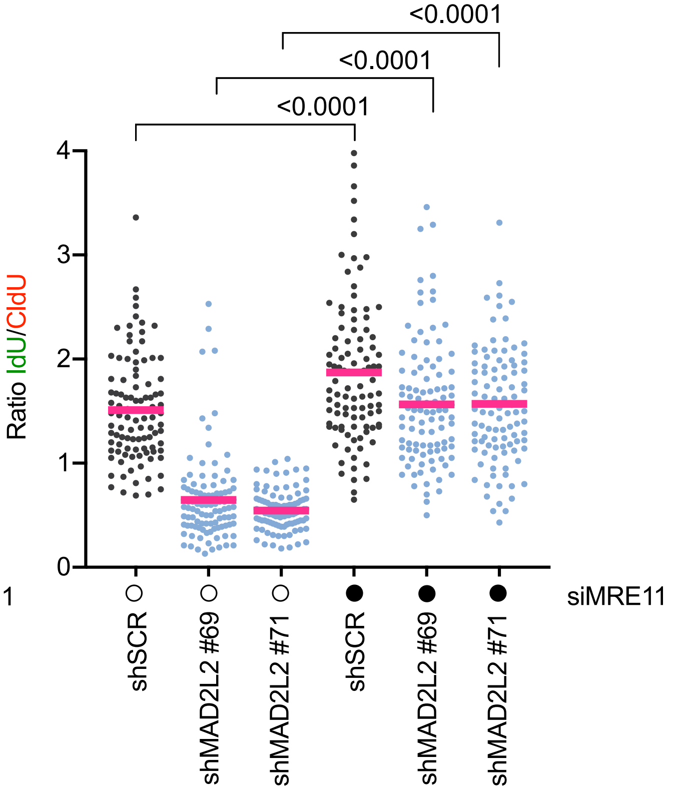

Additional replicates

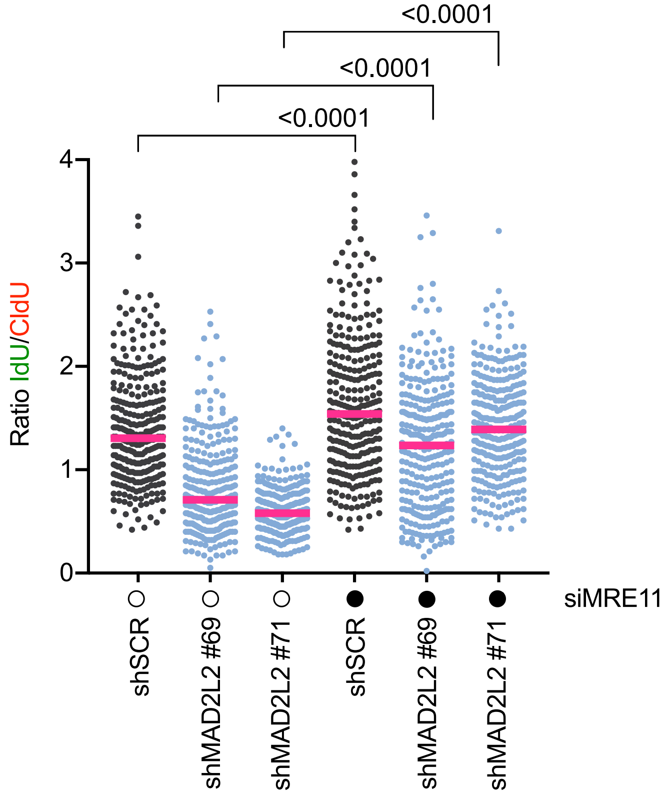

All replicates combined

Extended Data 1: Additional fiber replicates and combined fiber plots of all replicates

Extended Data 1j - related to Fig. 3c

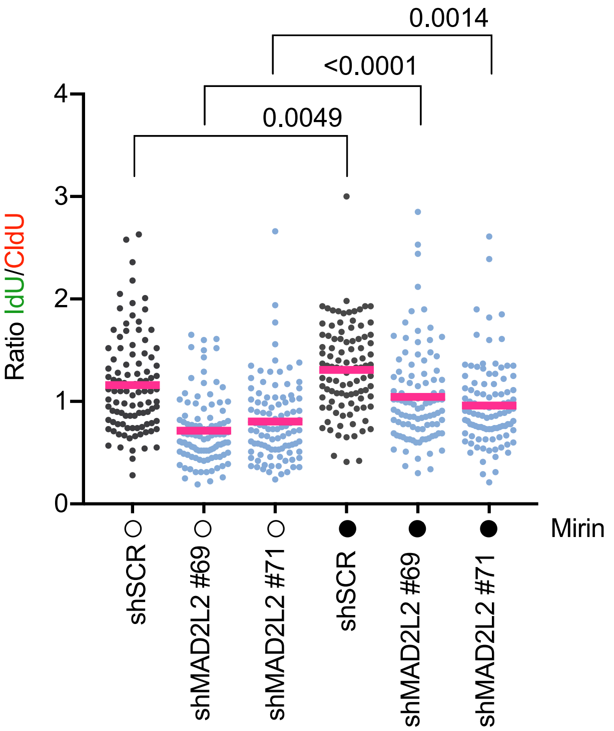

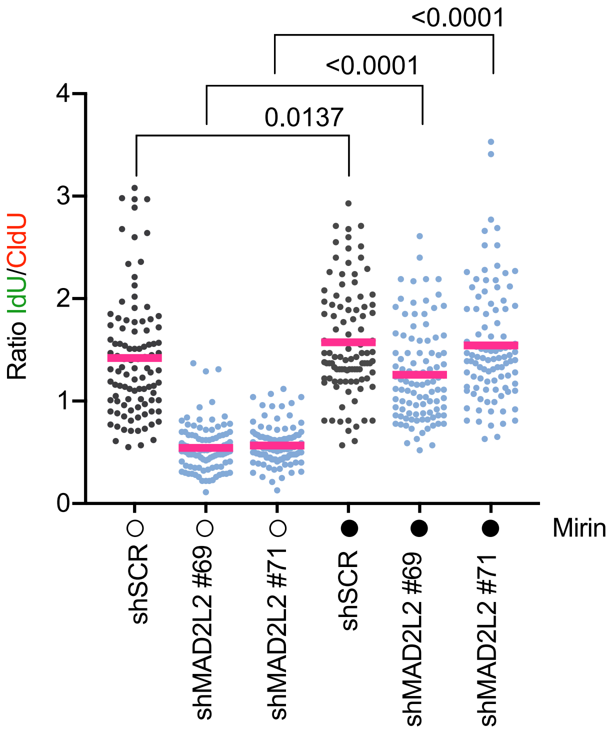

Additional replicates

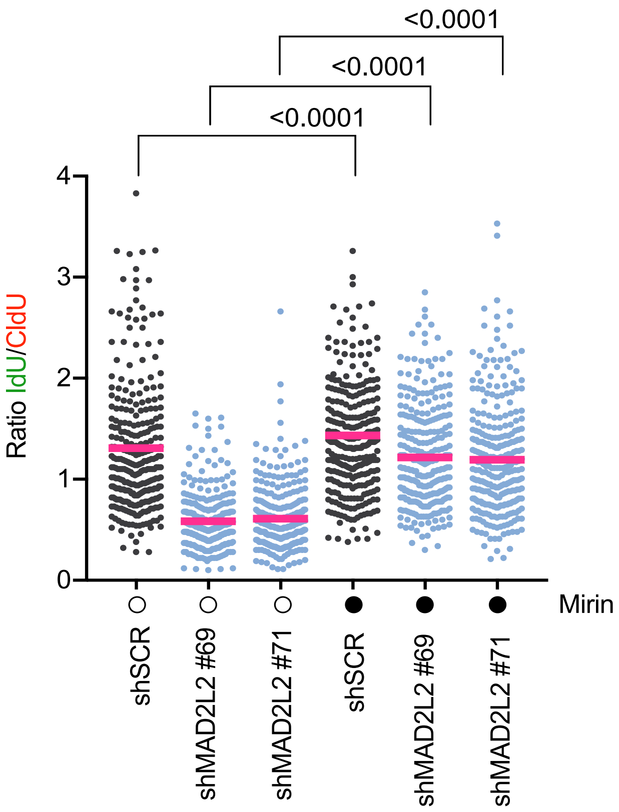

All replicates combined

Extended Data 1: Additional fiber replicates and combined fiber plots of all replicates

Extended Data 1k - related to Fig. 4d

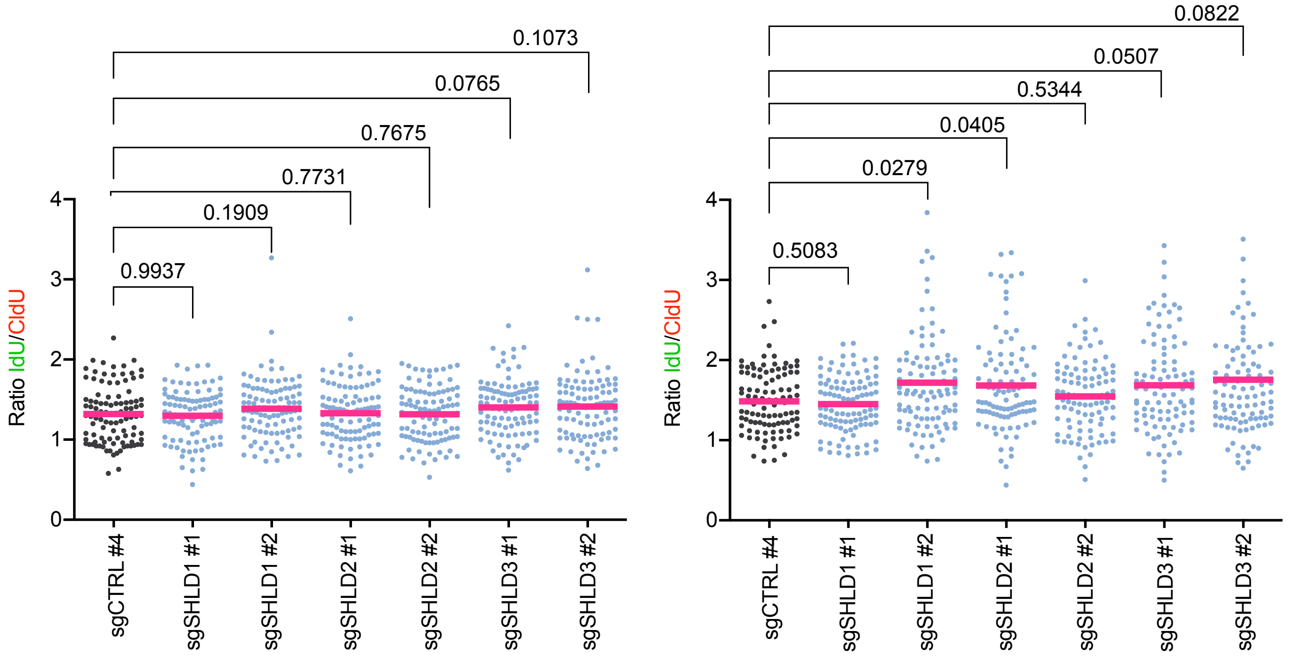

Additional replicates

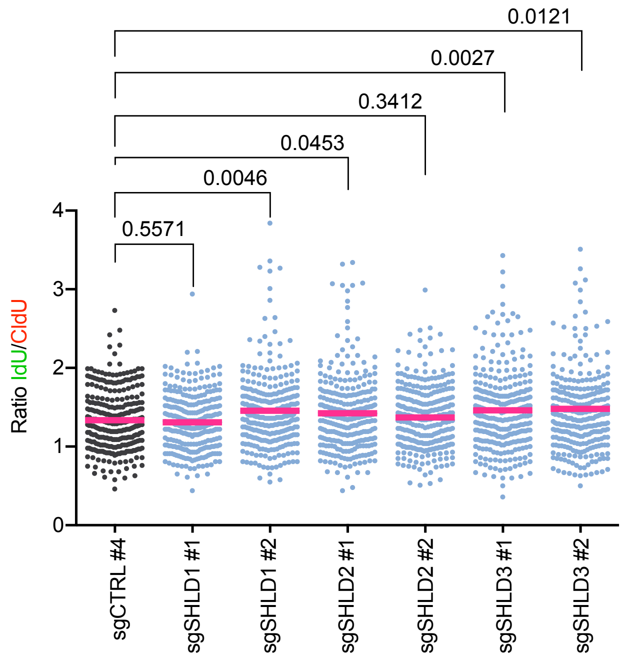

All replicates combined

Extended Data 1: Additional fiber replicates and combined fiber plots of all replicates

Extended Data 1l - related to Fig. 4e

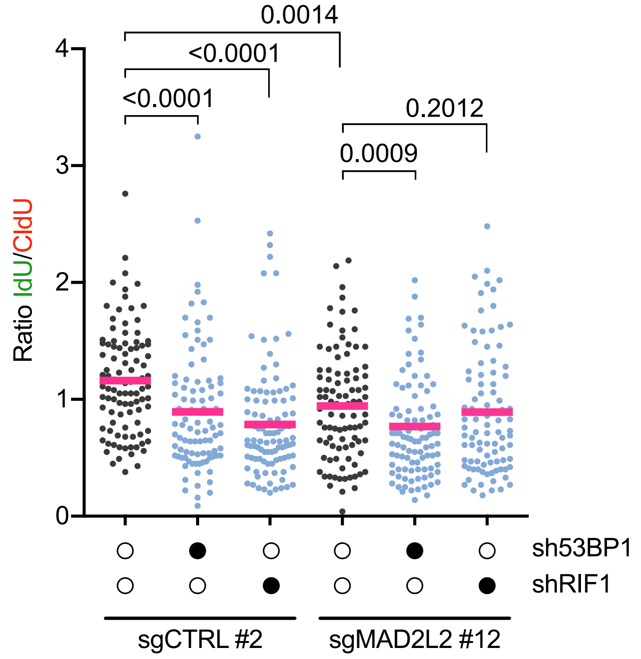

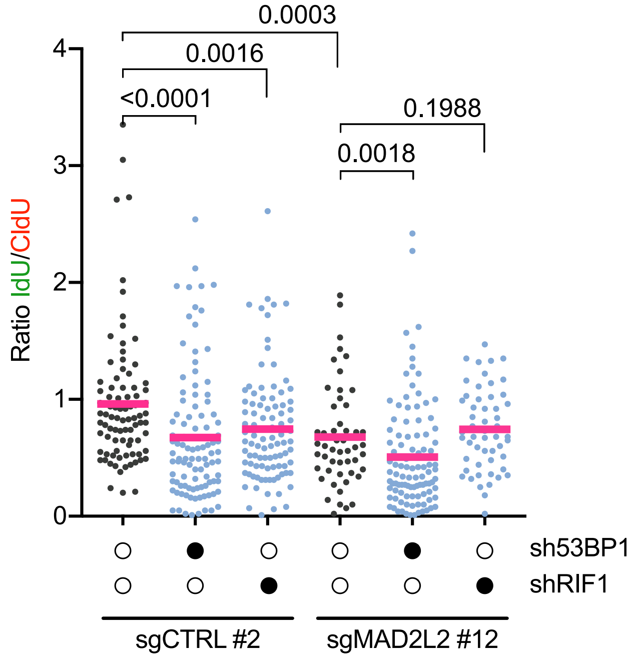

Additional replicates

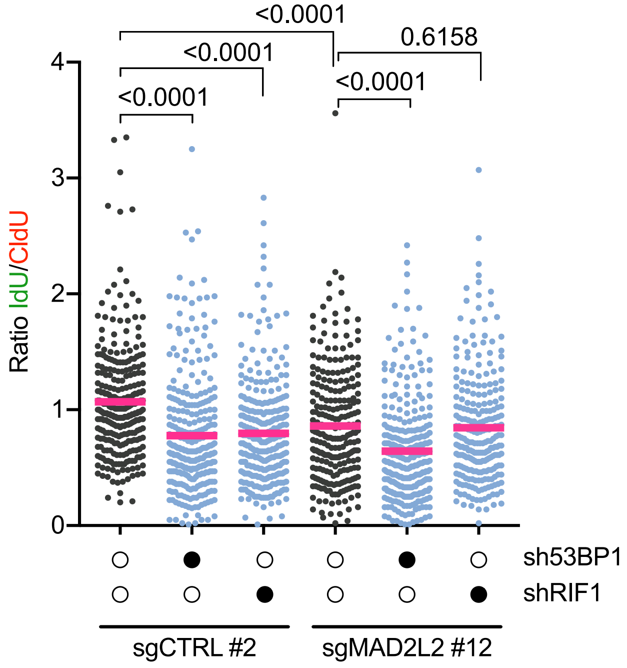

All replicates combined

Extended Data 1: Additional fiber replicates and combined fiber plots of all replicates

Extended Data 1m - related to Fig. 4f

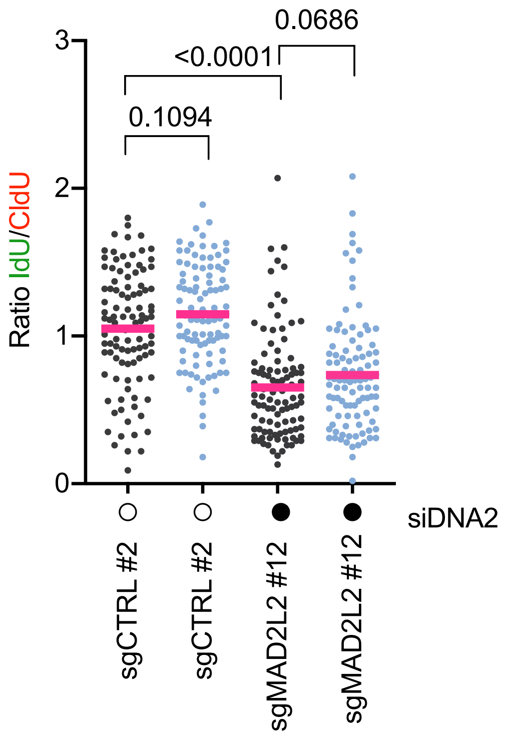

Additional replicate

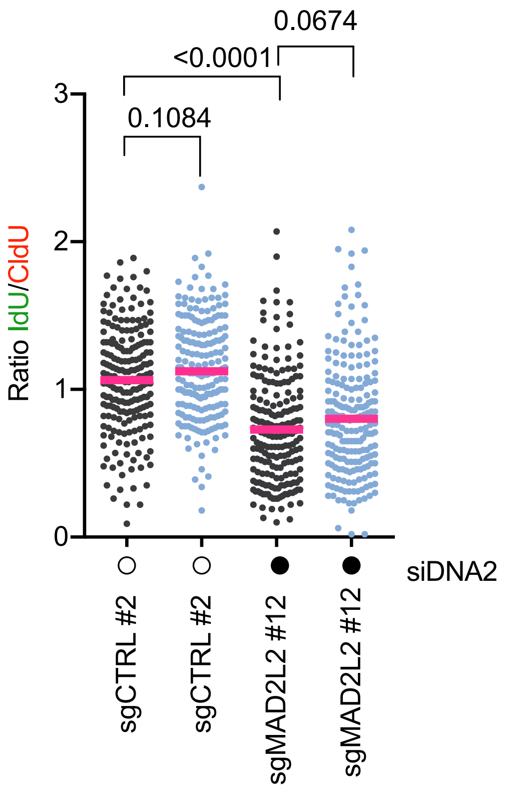

All replicates combined

Extended Data 1: Additional fiber replicates and combined fiber plots of all replicates

Extended Data 1n - related to Fig. S4b

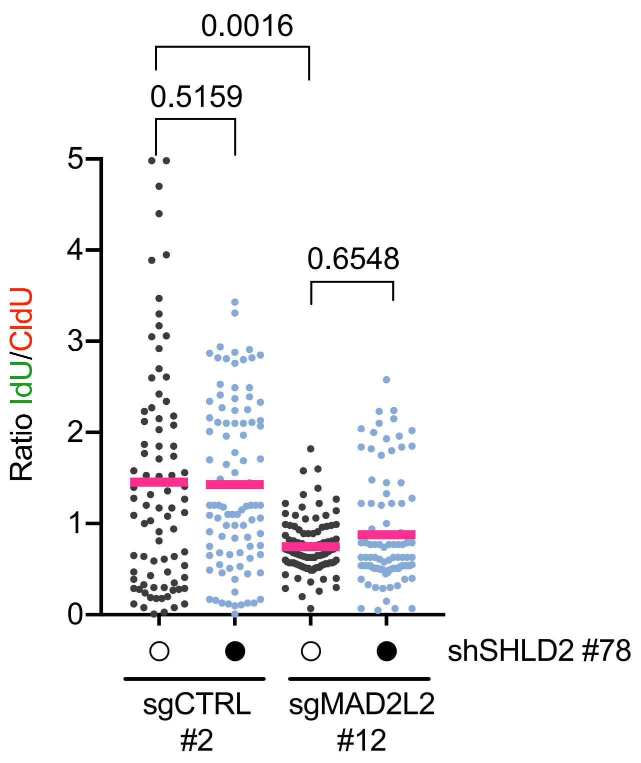

Additional replicate

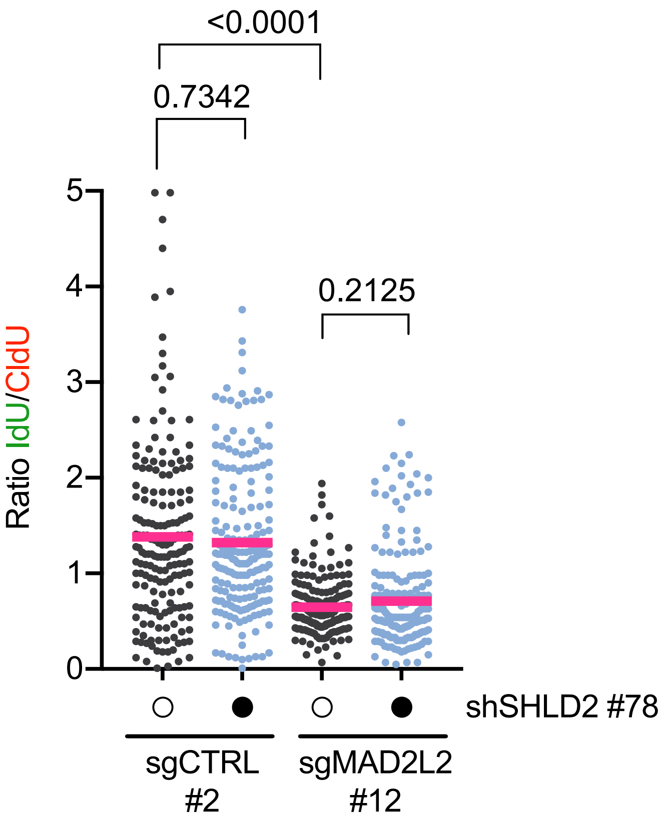

All replicates combined

Extended Data 1: Additional fiber replicates and combined fiber plots of all replicates

Extended Data 1o - related to Fig. 5c

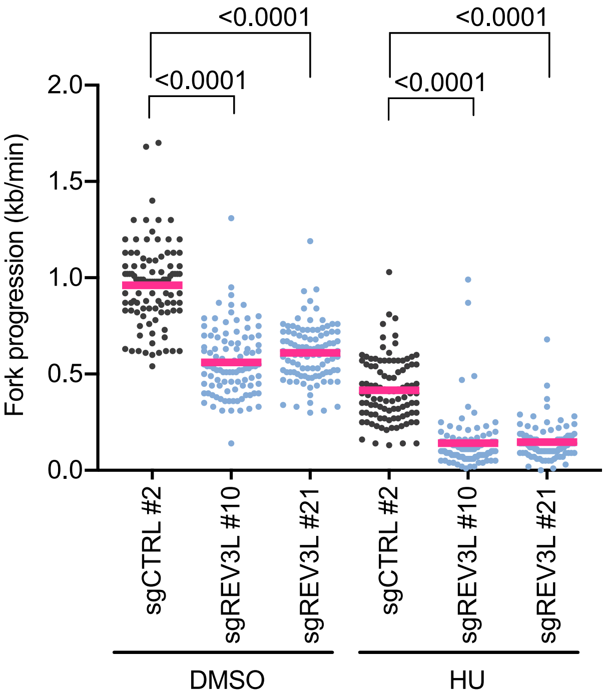

Additional replicate

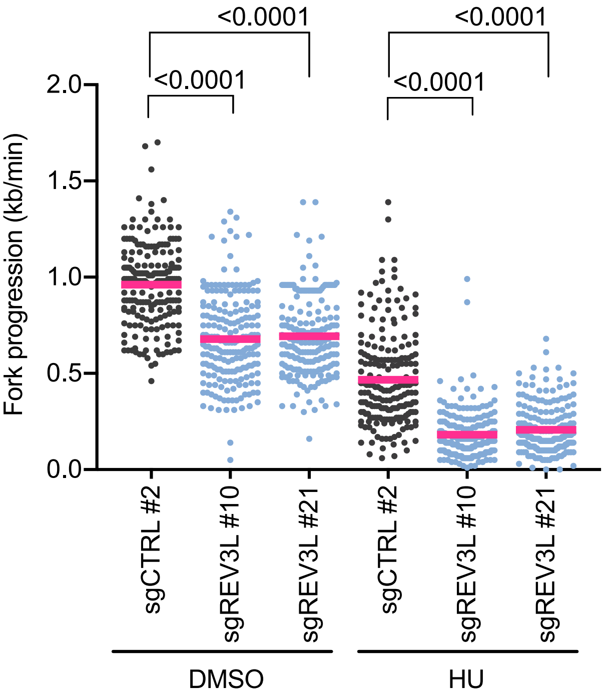

All replicates combined

Extended Data 1: Additional fiber replicates and combined fiber plots of all replicates

Extended Data 1p - related to Fig. 5d

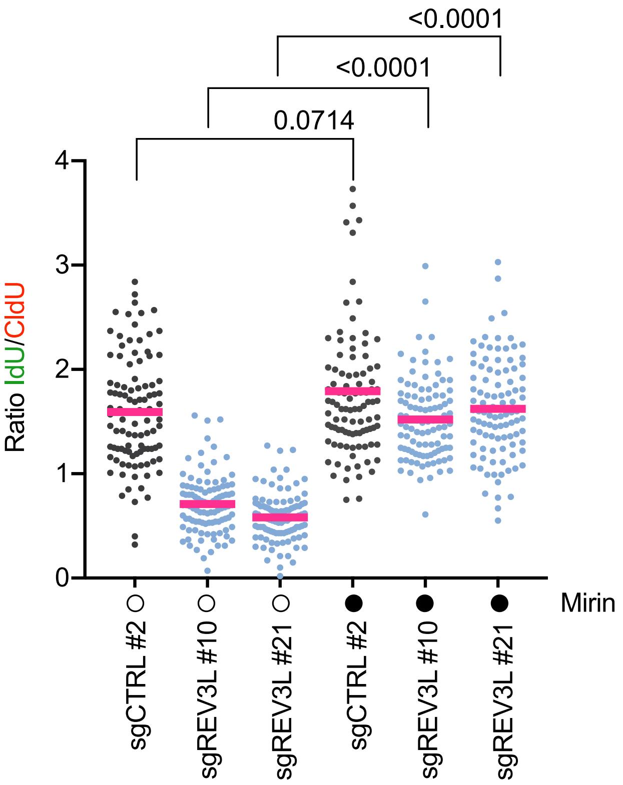

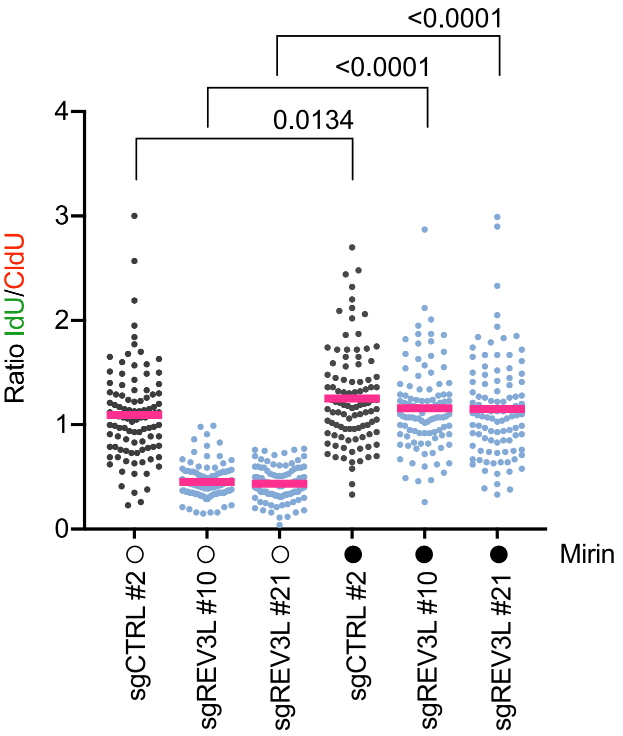

Additional replicates

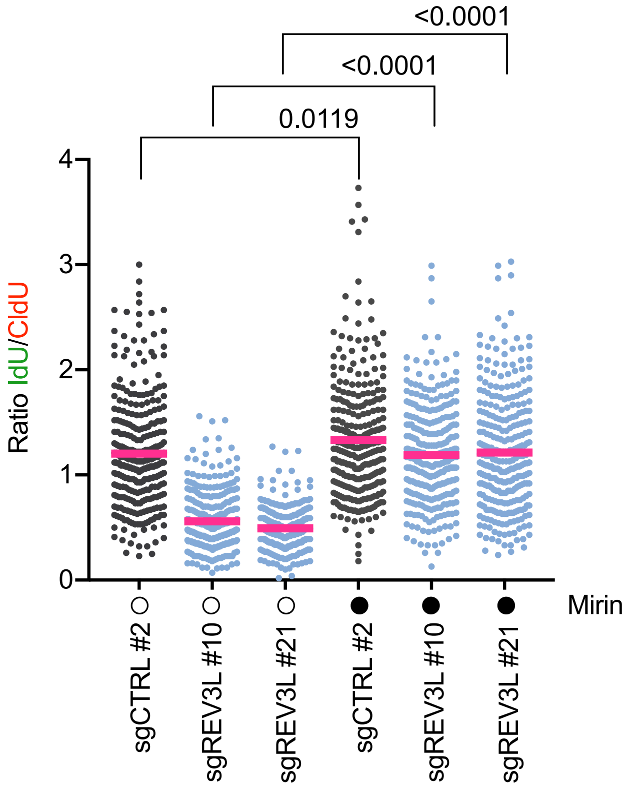

All replicates combined

Extended Data 1: Additional fiber replicates and combined fiber plots of all replicates

Extended Data 1q- related to Fig. 6a

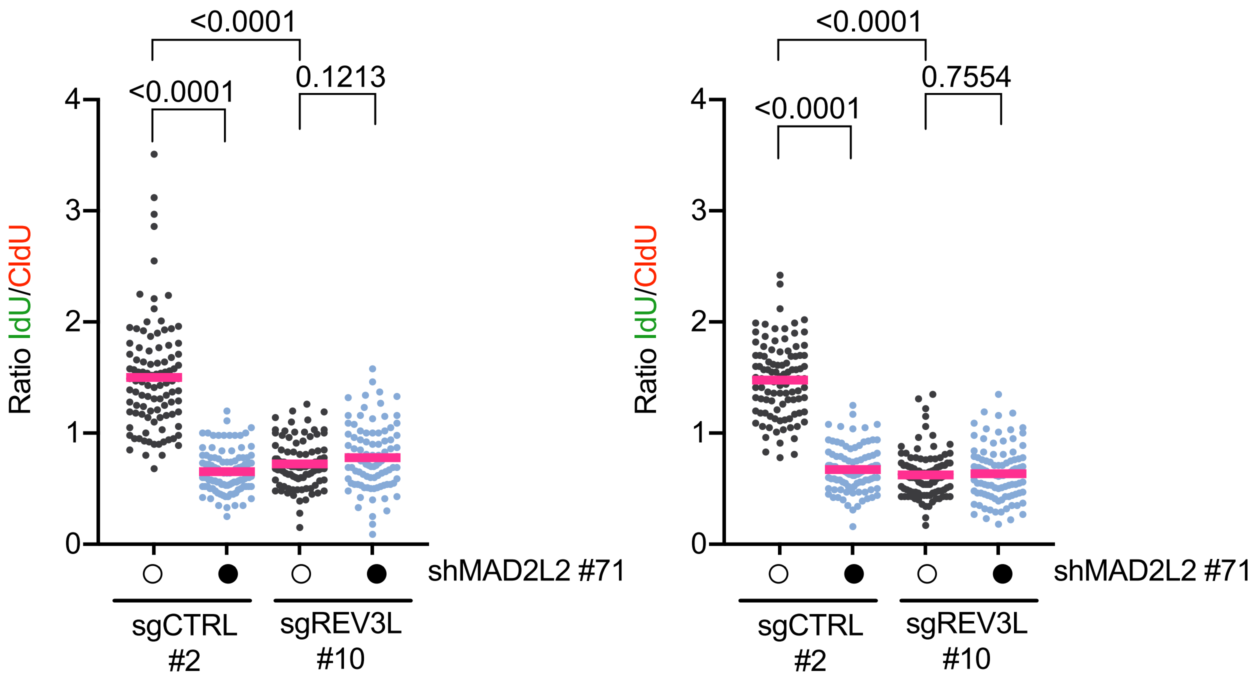

Additional replicates

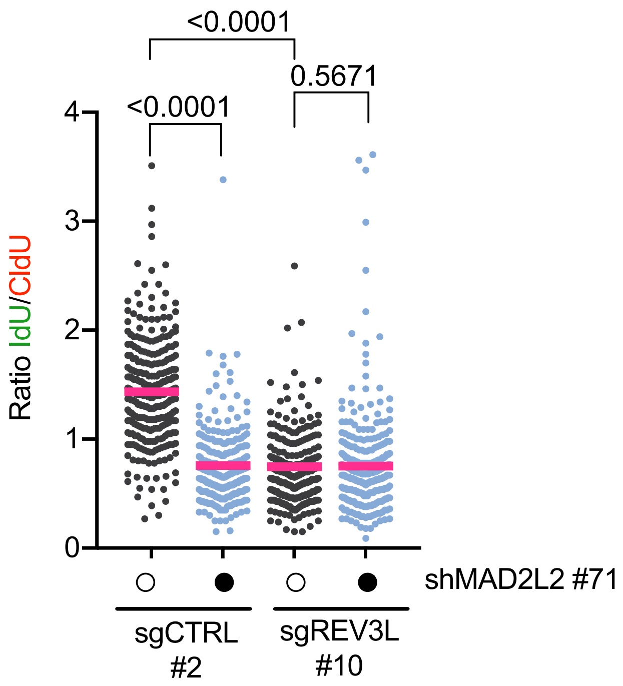

All replicates combined

Extended Data 1: Additional fiber replicates and combined fiber plots of all replicates

Extended Data 1r - related to Fig. 6e

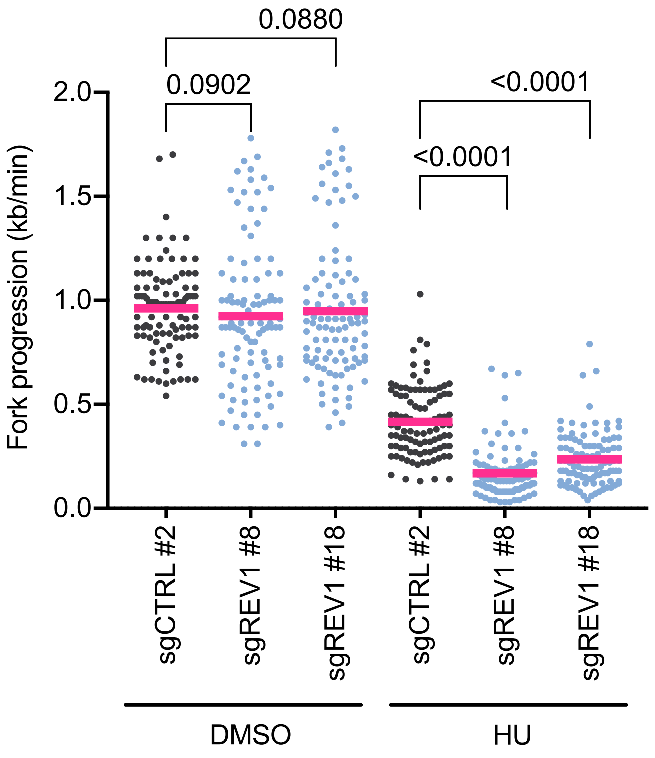

Additional replicate

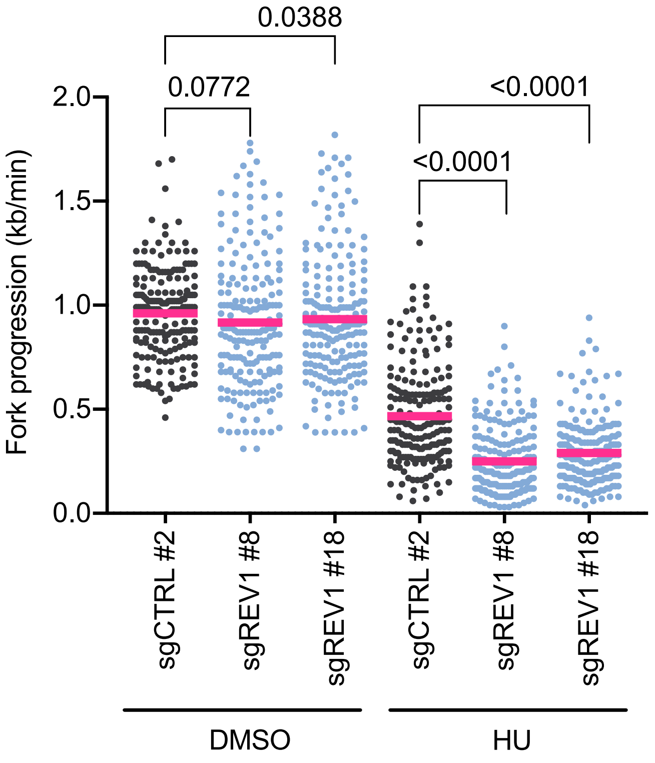

All replicates combined

Extended Data 1: Additional fiber replicates and combined fiber plots of all replicates

Extended Data 1s - related to Fig. 6f

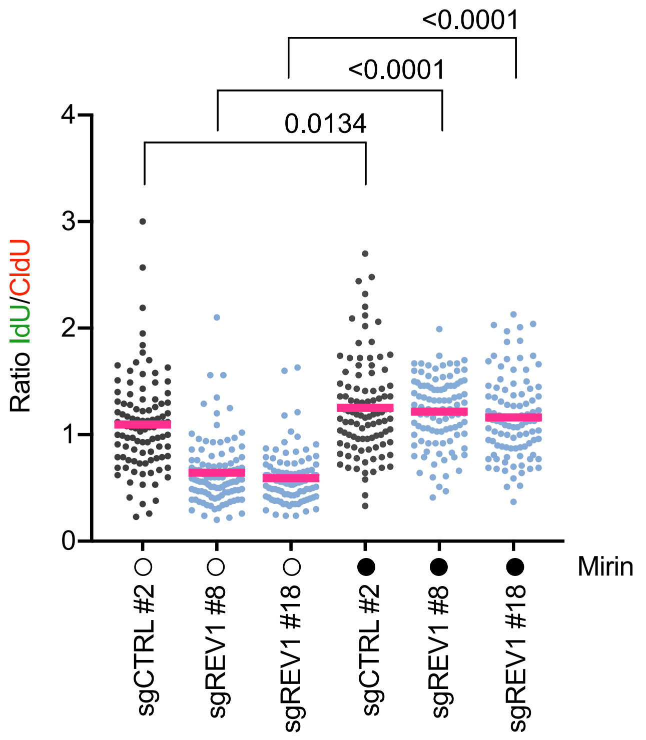

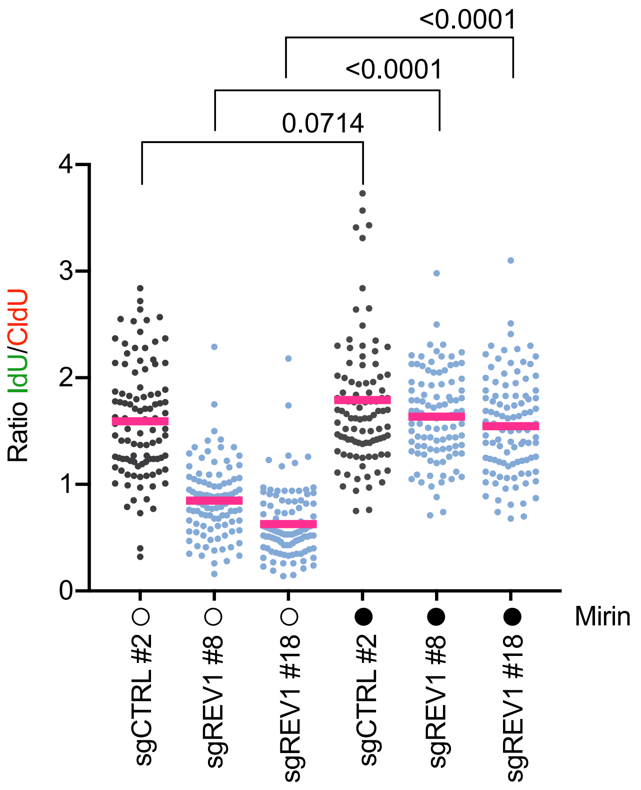

Additional replicates

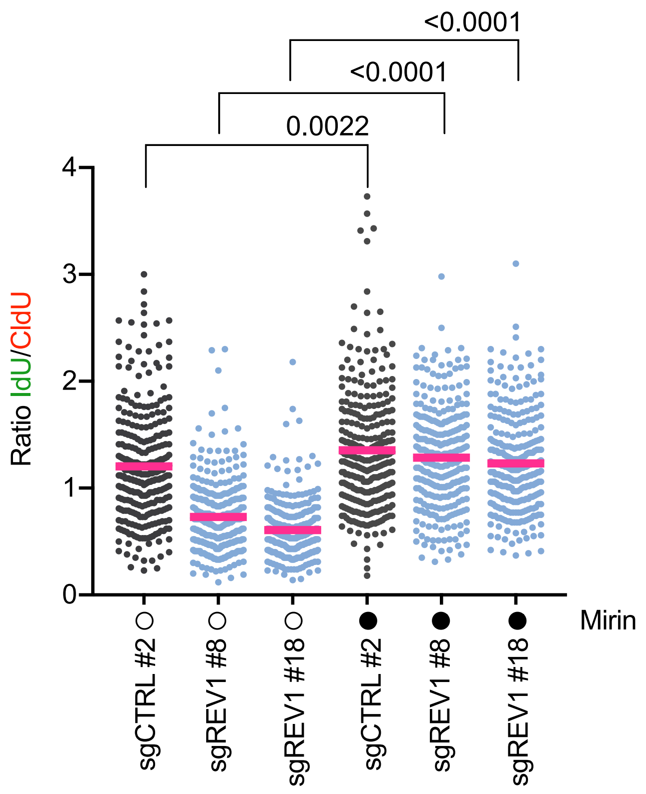

All replicates combined

Extended Data 1: Additional fiber replicates and combined fiber plots of all replicates

Extended Data 1t - related to Fig. 6h

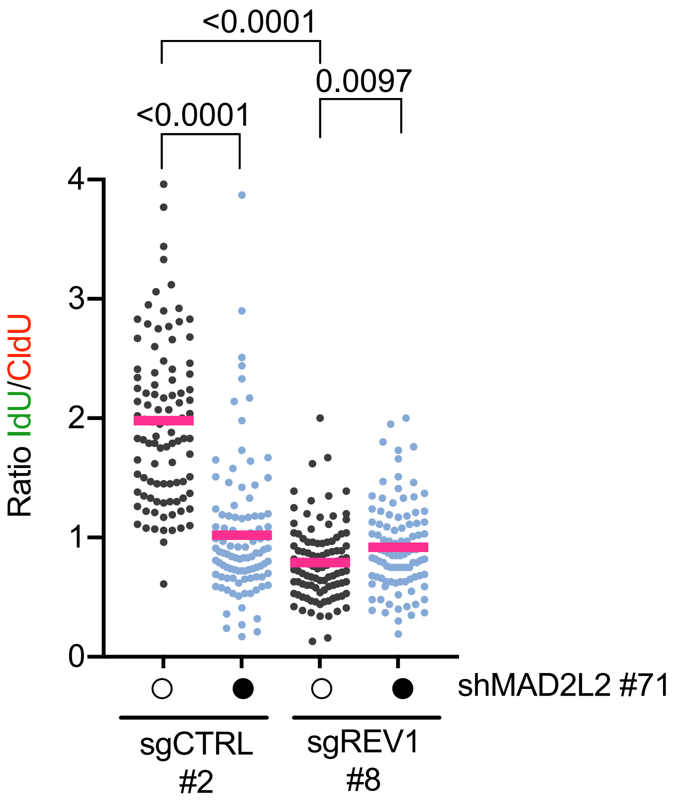

Additional replicate

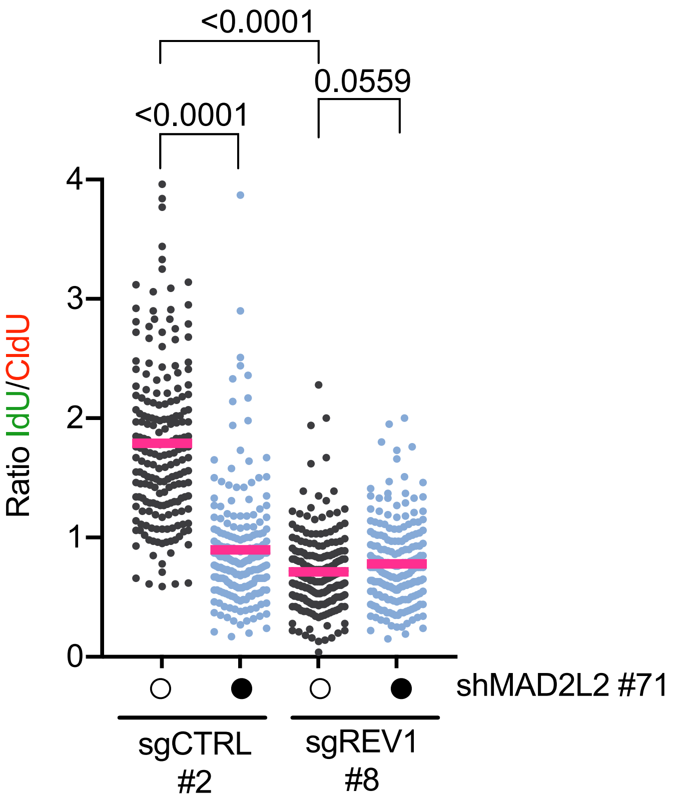

All replicates combined

Extended Data 1: Additional fiber replicates and combined fiber plots of all replicates

Extended Data 1u - related to Fig. 7a

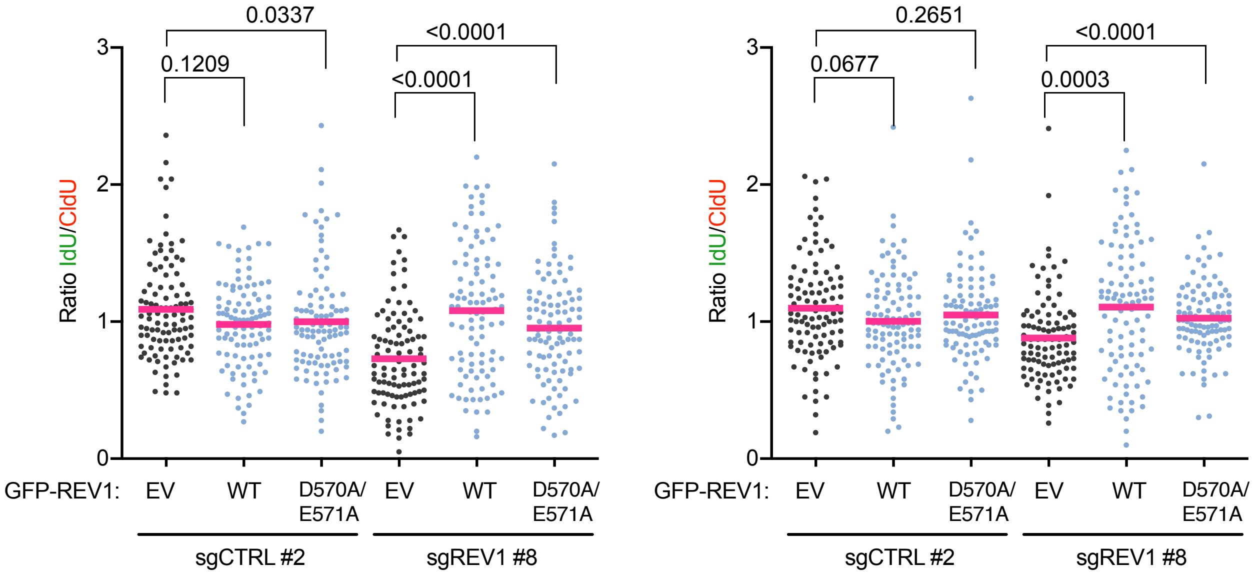

Additional replicates

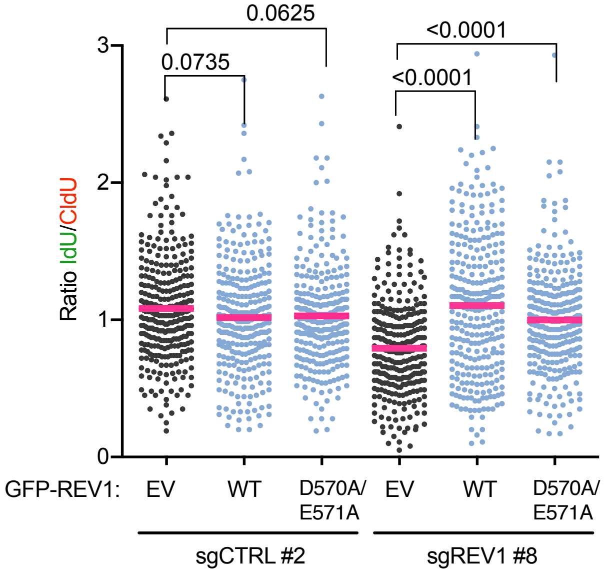

All replicates combined

Extended Data 1: Additional fiber replicates and combined fiber plots of all replicates

Extended Data 1v - related to Fig. 7b

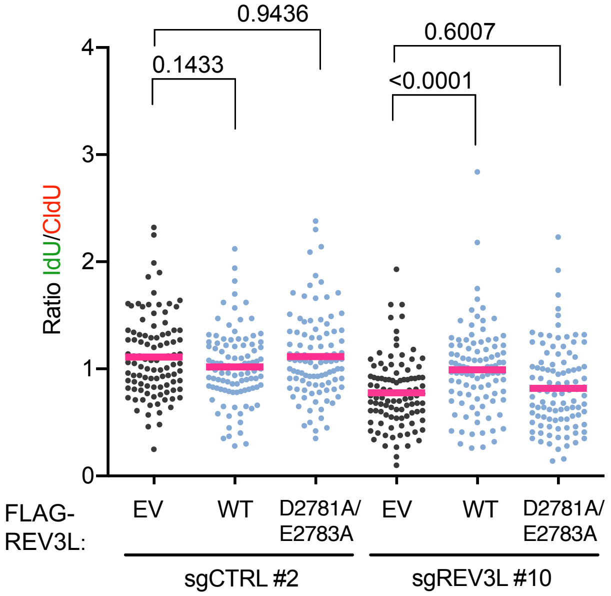

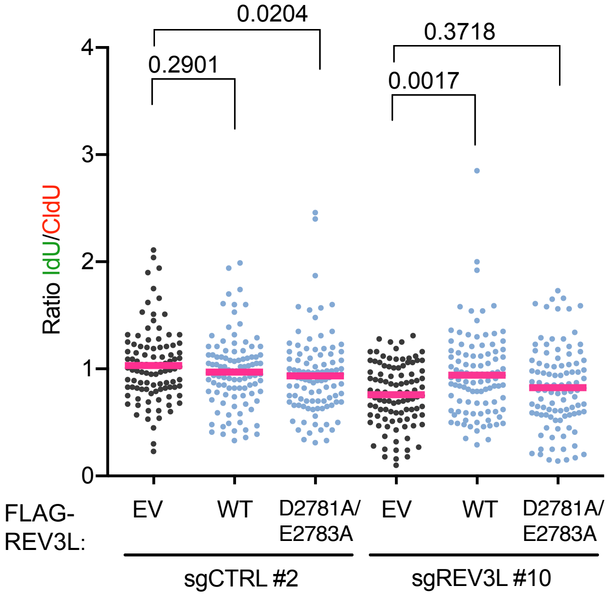

Additional replicates

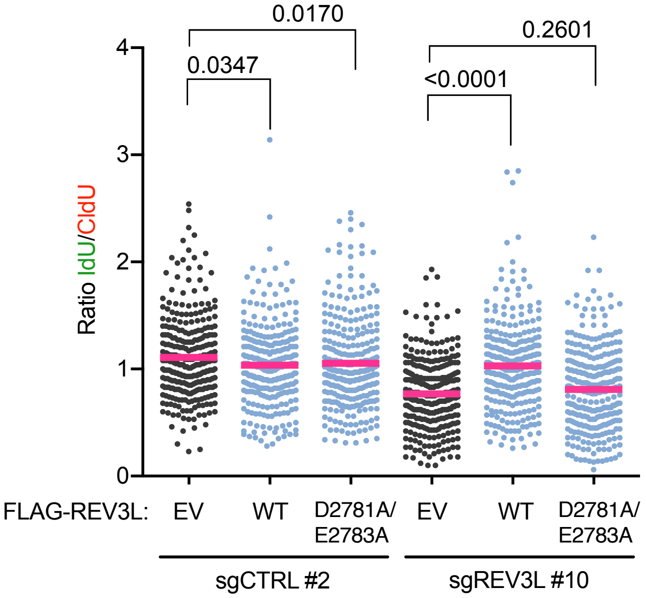

All replicates combined
