# Supplementary material for: MAD2L2 promotes replication fork protection and recovery in a shieldin-independent and REV3L-dependent manner
Source: Nat Commun. 2022 Sep 8;13:5167. doi: 10.1038/s41467-022-32861-5 (PMC9458726; doi:10.1038/s41467-022-32861-5)
Supplement: Supplementary file 3 — Description of Additional Supplementary Files [file 41467_2022_32861_MOESM3_ESM.pdf]

Description of Additional Supplementary Files

File Name: **Supplementary Data 1**

Description: An overview of the sgRNA and shRNA sequences used in this study (sgRNAs: tab 1; shRNAs: tab 2).

File Name: **Supplementary Data 2**

Description: An overview of the genomic PCR primers used in this study to check editing of sgRNAs (tab 1), a list of the primers used for RT-qPCR (tab 2), and a list of the primers used for site-directed mutagenesis (tab 3).

File Name: **Supplementary Data 3**

Description: Gene-editing efficiencies of the sgRNAs targeting *SHLD1* as determined by genome sequencing and TIDE analysis.
