# Supplementary material for: MAD2L2 promotes replication fork protection and recovery in a shieldin-independent and REV3L-dependent manner
Source: Nat Commun. 2022 Sep 8;13:5167. doi: 10.1038/s41467-022-32861-5 (PMC9458726; doi:10.1038/s41467-022-32861-5)
Supplement: Supplementary file 7 — Reporting Summary [file 41467_2022_32861_MOESM7_ESM.pdf]

nature portfolio

Corresponding author(s): J.J.L. Jacobs

Last updated by author(s): 08/2/2022

Reporting Summary

Nature Portfolio wishes to improve the reproducibility of the work that we publish. This form provides structure for consistency and transparency in reporting. For further information on Nature Portfolio policies, see our Editorial Policies and the Editorial Policy Checklist.

Statistics

For all statistical analyses, confirm that the following items are present in the figure legend, table legend, main text, or Methods section.

n/a	Confirmed	
☐	☒	The exact sample size (*n*) for each experimental group/condition, given as a discrete number and unit of measurement
☐	☒	A statement on whether measurements were taken from distinct samples or whether the same sample was measured repeatedly
☐	☒	The statistical test(s) used AND whether they are one- or two-sided<br>*Only common tests should be described solely by name; describe more complex techniques in the Methods section.*
☒	☐	A description of all covariates tested
☐	☒	A description of any assumptions or corrections, such as tests of normality and adjustment for multiple comparisons
☐	☒	A full description of the statistical parameters including central tendency (e.g. means) or other basic estimates (e.g. regression coefficient) AND variation (e.g. standard deviation) or associated estimates of uncertainty (e.g. confidence intervals)
☐	☒	For null hypothesis testing, the test statistic (e.g. *F*, *t*, *r*) with confidence intervals, effect sizes, degrees of freedom and *P* value noted<br>*Give P values as exact values whenever suitable.*
☒	☐	For Bayesian analysis, information on the choice of priors and Markov chain Monte Carlo settings
☒	☐	For hierarchical and complex designs, identification of the appropriate level for tests and full reporting of outcomes
☒	☐	Estimates of effect sizes (e.g. Cohen's *d*, Pearson's *r*), indicating how they were calculated

Our web collection on statistics for biologists contains articles on many of the points above.

Software and code

Policy information about availability of computer code

Data collection

1. Syngene G:BOX (Syngene)
2. IncuCyte ZOOM (Essen Bioscience).
3. AxioObserver Z1 microscope (Zeiss)
4. LSR II Flow Cytometer (BD Biosciences)
5. Metafer4/MSearch (Metasystems) on AxioImagerZ2 microscope (Zeiss)
6. Infinite M200pro (TECAN)
7. Fortessa analyser (BD Biosciences)
8. QuantStudio 6 Flex real-time PCR system (Thermo Fisher Scientific)
9. Odyssey CLx infrared scanning device (LI-COR)
10. Zeiss LSM 980 confocal with Airyscan2

Data analysis

1. GeneSys (version 1.8.5.0)
2. IncuCyte (version 2018A)
3. ZEN (version 2.6)
4. FlowJo (version 10.7.1)
5. GraphPad Prism (version 9)
6. Fiji (version 1.53a, 1.53q and 2.0)
7. Microsoft Excel (version 16.16.27)
8. Image Studio Lite LI-COR (version 5.2.5)
9. CASP (http://www.casp.of.pl, version 1.2.3 beta 2)
10. TIDE (version 3.3.0)

For manuscripts utilizing custom algorithms or software that are central to the research but not yet described in published literature, software must be made available to editors and reviewers. We strongly encourage code deposition in a community repository (e.g. GitHub). See the Nature Portfolio guidelines for submitting code & software for further information.

Data

Policy information about availability of data

All manuscripts must include a data availability statement. This statement should provide the following information, where applicable:

- Accession codes, unique identifiers, or web links for publicly available datasets
- A description of any restrictions on data availability
- For clinical datasets or third party data, please ensure that the statement adheres to our policy

All data generated or analyzed during this study are included in this published article (and its Supplementary information files). Source data are provided in the Source data file, which includes all uncropped blots and data shown in graphs throughout the manuscript.

Field-specific reporting

Please select the one below that is the best fit for your research. If you are not sure, read the appropriate sections before making your selection.

☒ Life sciences ☐ Behavioural & social sciences ☐ Ecological, evolutionary & environmental sciences

For a reference copy of the document with all sections, see nature.com/documents/nr-reporting-summary-flat.pdf

Life sciences study design

All studies must disclose on these points even when the disclosure is negative.

Sample size	No sample-size calculations were performed. The number of biological replicates is mentioned in the figure legends and the corresponding data point for each individual biological replicate are shown in the graphs. We included information on the (range in the) number of cells used for immunofluorescence analysis in the methods.<br>1. For metaphase spread analysis to address chromosomal aberrations, we analyzed 50 metaphase spreads per condition per experiment. Analysis of 50 metaphases per condition and replicate is sufficient to obtain a reliable mean.<br>2. For immunofluorescence experiments, we examined a minimum of 100 cells per condition per experiment, with the exception of the neutral comet assays, in which at least 50 cells were analyzed. Analysis of >100 cells for immunofluorescence and >50 cells for the neutral comet assay, respectively, was sufficient to obtain a representative measure (reliable mean).
Data exclusions	No data was excluded.
Replication	Experiments were performed at least in duplicate, but mostly in triplicate to asses the reproducibility. All attempts at reproduction were successful. Graphs are represented as dot plots showing the spread among the replicates of the individual experiments. Standard errors are included in graphs that contain more than two replicates and that indicate the variation between replicates. Number of biological replicates for each experiment are indicated in the respective figure legends.
Randomization	There was no allocation of test subjects for any of the experiments, thus randomization was not applicable to our study. Nonetheless, no specific order of processing samples was maintained between experiments. Experiments were performed with cell lines that were seeded randomly for different treatments.
Blinding	Genotypes were blinded prior to manual scoring of the samples for fiber analysis. Blinding for other approaches used in our manuscript, including western blots and siRNA/inhibitor treatments, has not been applied because it is not feasible for these experiments.

Reporting for specific materials, systems and methods

We require information from authors about some types of materials, experimental systems and methods used in many studies. Here, indicate whether each material, system or method listed is relevant to your study. If you are not sure if a list item applies to your research, read the appropriate section before selecting a response.

Materials & experimental systems

n/a	Involved in the study	
☐	☒	Antibodies
☐	☒	Eukaryotic cell lines
☒	☐	Palaeontology and archaeology
☒	☐	Animals and other organisms
☒	☐	Human research participants
☒	☐	Clinical data
☒	☐	Dual use research of concern

Methods

n/a	Involved in the study	
☒	☐	ChIP-seq
☐	☒	Flow cytometry
☒	☐	MRI-based neuroimaging

Antibodies

Antibodies used

For an overview of antibodies used, see material and methods.
Primary antibodies:
1. MAD2L2, sc135977, Santa-Cruz, 1:1,000
2. REV3L, GTX100153, GeneTex, 1:500
3. REV1, OTI1E12, MA5-27493, Thermo Fisher Scientific, 1:500
4. HSP90, sc-7947, Santa Cruz, 1:5,000
5. Tubulin, T6557, Sigma, 1:1,000
6. H2AX, 5636-I, Millipore, 1:1,000
7. GFP, sc-8334, Santa Cruz, 1:1000
8. FLAG, F1804, Sigma, 1:1000
9. HDAC1, PA1-860, Thermo Fisher Scientific, 1:1000
10. GAPDH, PA1-987, Thermo Fisher Scientific, 1:10000
11. Biotin, Jackson Immunoresearch, 200-002-211, 1:1,000
12. MRE11, Novus, NB100-142, 1:100
13. anti-BrdU, Abcam, Ab6326, 1:400
14. anti-BrdU, B44, Becton Dickinson, 347580, 1:400

Secondary antibodies:
1. Alexa Fluor 488 goat anti-mouse IgG, A11029, Thermo Fisher Scientific, 1:150
2. Alexa Fluor 568 goat anti-rat IgG, A11077, Thermo Fisher Scientific, 1:150
3. Goat anti-rabbit IgG HRP, G21234, Thermo Fisher Scientific, 1:7,500
4. Goat anti-mouse IgG HRP, G21040, Thermo Fisher Scientific, 1:7,500
5. IRDye 800CW donkey anti-rabbit, IgG 926-32213, Li-cor, 1:10000
6. IRDye 680RD donkey anti-mouse, IgG 926-68072, Li-cor, 1:10000

Validation

The following antibodies were validated as stated on suppliers website:
-anti-REV3L (rabbit polyclonal,GTX100153, GeneTex) validation stated on suppliers website: https://www.genetex.com/Product/Detail/REV3L-antibody-C3-C-term/GTX100153#document
-anti-H2AX (mouse monoclonal, 05-636, Millipore) validation stated on suppliers website: https://www.merckmillipore.com/NL/en/product/Anti-phospho-Histone-H2A.X-Ser139-Antibody-clone-JBW301,MM_NF-05-636?cid=BI-XX-BRC-A-NANT-ANTI-B057-1308
-anti-REV1 (mouse monoclonal, OTI1E12, MA5-27493, Thermo Fisher Scientific) no information on validation is provided by the supplier (see below for additional validation information)
-anti-GFP (rabbit polyclonal, sc-8334, Santa Cruz) validation stated on suppliers website: https://datasheets.scbt.com/sc-8334.pdf
-anti-FLAG (mouse monoclonal, F1804, Sigma) validation stated on suppliers website: https://www.sigmaaldrich.com/deepweb/assets/sigmaaldrich/product/documents/119/160/f1804bul-mk.pdf
-anti-HDAC1 (rabbit polyclonal, PA1-860, Thermo Fisher Scientific) validation stated on suppliers website: https://www.thermofisher.com/antibody/product/HDAC1-Antibody-Polyclonal/PA1-860
-anti-GAPDH (rabbit polyclonal, PA1-987, Thermo Fisher Scientific) validation stated on suppliers website:https://www.thermofisher.com/antibody/product/GAPDH-Antibody-Polyclonal/PA1-987
-anti-MRE11 (rabbit polyclonal, NB100-142, Novus) validation stated on suppliers website: https://www.novusbio.com/products/mre11-antibody_nb100-142
-anti-BrdU (rat monoclonal, Ab6326, Abcam) validation stated on suppliers website: https://www.abcam.com/brdu-antibody-bu175-icr1-proliferation-marker-ab6326.html
-anti-BrdU(mouse monoclonal, B44, 347580, Becton Dickinson) validation stated on suppliers website: https://www.bdbiosciences.com/content/bdb/paths/generate-tds-document.us.347580.pdf

The following antibodies were additionally validated by us in knockdown/knockout cells: MAD2L2 (mouse monoclonal, sc135977, Santa Cruz), REV3L (rabbit polyclonal, GTX100153, GeneTex), REV1 (mouse monoclonal, OTI1E12, Thermo Fisher Scientific).

The following antibodies are commonly used loading controls: HSP90 (rabbit polyclonal, sc7947, Santa Cruz), Tubulin (mouse monoclonal, T6557, Sigma), HDAC1 (rabbit polyclonal, PA1-860, Thermo Fisher Scientific), GAPDH (rabbit polyclonal, PA1-987, Thermo Fisher Scientific).

For the anti-Biotin (mouse monoclonal, 200-002-211, Jackson Immunoresearch), the information on validation by the supplier is no longer accessible, but several studies have validated the specificity of the antibody (PMID:28410996, PMID:29053959, PMID:29937342, PMID:30753836, PMID:32139584).

Eukaryotic cell lines

Policy information about cell lines

Cell line source(s)

1. HeLa, BJ-hTERT, RPE1-hTERT, and 293Ts originate from ATCC.
2. RPE1-hTERT were a gift from D. Durocher at The Lunenfeld-Tanenbaum Research Institute and are described before (Zimmerman M. et al, Nature, 2018). RPE1-hTERT were originally purchased from ATCC.

Authentication

None of the cell lines used were authenticated by us.

Mycoplasma contamination

All cell lines are routinely tested negative for mycoplasma contamination.

Commonly misidentified lines
(See ICLAC register)

No commonly misidentified cell lines were used in this study.

Flow Cytometry

Plots

Confirm that:

☒ The axis labels state the marker and fluorochrome used (e.g. CD4-FITC).

☒ The axis scales are clearly visible. Include numbers along axes only for bottom left plot of group (a 'group' is an analysis of identical markers).

☒ All plots are contour plots with outliers or pseudocolor plots.

☒ A numerical value for number of cells or percentage (with statistics) is provided.

Methodology

Sample preparation	Sample preparation is specified in the material and methods.<br>Cells were collected by trypsinization, washed with PBS, fixed in ethanol, and incubated with RNase A and propidium iodide (PI). At least 10,000 cells per sample were recorded.
Instrument	1. Fortessa analyser (BD Biosciences)<br>2. LSR II Flow Cytometer (BD Biosciences)
Software	FlowJo (version 10.7.1)
Cell population abundance	No cell sorting was used in this study.
Gating strategy	Initial gates (FSC-A/SSC-A) to discriminate cells from debris; then cells were gated for single cells (FSC-H/FSC-W). The singlet, live cells were analyzed for PI fluorescence to identify the proportion of cells that in are in each cell cycle phase.

☒ Tick this box to confirm that a figure exemplifying the gating strategy is provided in the Supplementary Information.
